# Fat sensory cues in early life program central response to food and obesity

**DOI:** 10.1038/s42255-025-01405-8

**Published:** 2025-12-01

**Authors:** Laura Casanueva Reimon, Ayden Gouveia, André Carvalho, Joscha N. Schmehr, Mouna El Mehdi, Rolando D. Moreira-Soto, Carlos G. Ardanaz, Janice Bulk, Lionel Rigoux, Paul Klemm, Anna Lena Cremer, Frederik Dethloff, Yvonne Hinze, Heiko Backes, Patrick Giavalisco, Sophie M. Steculorum

**Affiliations:** 1 Max Planck Institute for Metabolism Research, Max Planck Research Group Neurocircuit Wiring and Function, Cologne, Germany; 2 Excellence Cluster on Cellular Stress Responses in Aging-Associated Diseases (CECAD), University of Cologne, Cologne, Germany; 3 Max Planck Institute for Chemical Ecology, Department of Evolutionary Neuroethology, Jena, Germany; 4 Universidad de Costa Rica, Centro de Investigación en Enfermedades Tropicales, Facultad de Microbiología, San José, Costa Rica; 5 Max Planck Institute for Metabolism Research, Translational Neurocircuitry Group, Cologne, Germany; 6 Max Planck Institute for Metabolism Research, Neuronal Control of Metabolism Group, Cologne, Germany; 7 Max Planck Institute for Metabolism Research, Multimodal Imaging Group, Cologne, Germany; 8 Max Planck Institute for Biology of Ageing, Metabolomics Core Facility, Cologne, Germany; 9 German Center of Diabetes Research (DZD), Neuherberg, Germany

**Keywords:** Obesity, Feeding behaviour

## Abstract

Maternal obesity predisposes offspring to metabolic diseases. Here, we show that non-nutritive sensory components of a high-fat diet (HFD), beyond its hypercaloric, obesogenic effects, are sufficient to alter metabolic health in the offspring. To dissociate the caloric and sensory components of HFD, we fed dams a bacon-flavoured diet, isonutritional to a normal chow diet but enriched with fat-related odours. Offspring exposed to these fat-related odours during development display metabolic inflexibility and increased adiposity when fed HFD in adulthood independently of maternal metabolic health. Developmental exposure to fat-related odours shifts mesolimbic dopaminergic circuits and Agouti-related peptide (AgRP) hunger neurons’ responses to phenocopy those of obese mice, including a desensitization of AgRP neurons to dietary fat. While neither neonatal optogenetic activation of sensory circuits nor passive exposure to fat-related odours is sufficient to alter metabolic responses to HFD, coupling optogenetic stimulation of sensory circuits with caloric intake exacerbates obesity. Collectively, we report that fat-related sensory cues during development act as signals that can prime central responses to food cues and whole-body metabolism regulation.

## Abstract (web summary)

Non-nutritive sensory components of high-fat diet, such as bacon flavour, are sufficient to impair metabolic health in offspring in mice.

## Main

Exposure to a maternal hypercaloric, hyperlipidaemic diet during development is a major risk factor for life-long obesity and metabolic syndrome^1–5^. Maternal obesity and consumption of calorie-rich high-fat foods predispose offspring to a wide array of metabolic and behavioural impairments, including adiposity and insulin resistance, and shift food preference towards increased intake of highly palatable foods^1–5^. Extensive research has shown that the metabolic consequences of maternal high-fat diet (HFD) consumption, such as excess gestational weight gain, adiposity and insulin resistance, are considered key contributors to the metabolic programming of the offspring^1–5^. However, outcomes of metabolic programming can occur in lean HFD-fed dams independent of maternal adiposity or insulin resistance^1–3,6–8^, suggesting that the heightened risk of obesity development in the offspring is not solely triggered by maternal obesity or nutritive components of HFD per se. It is unknown which components of HFD, aside from its nutritive effects, can trigger metabolic programming.

Food is not only composed of nutrients, but also contains non-nutritive sensory components such as volatile odours. Although the hypercaloric component of HFD has been a major focus of interest, HFD carries a distinct chemosensory signature. During development, fetuses and newborns are exposed to both nutritive and non-nutritive sensory food signals where volatile odours from the mother’s diet are directly transferred to the amniotic fluid and milk^9–14^. Perinatal olfactory experiences foster sensory memories upon which the organism relies throughout life to make food choices and dictate feeding habits until adulthood towards a preference for the odours encountered during development^12,13,15–19^. To date, the consequences of developmental exposure to non-nutritive sensory food cues of HFD on life-long regulation of the central representation, preference, intake and metabolic response to HFD remain elusive.

While the role of the sensory environment during development is yet to be defined, food sensory perception has emerged as a regulator of key neural circuits that control feeding behaviour and whole-body metabolism in adulthood^20–29^. Here, we explored the influence of fat-related sensory cues during development, independent of the nutritive content, on the regulation of the central responses to food cues, metabolic health and obesity susceptibility during adulthood.

## A model for developmental exposure to fat-related sensory cues

**Fig. 1:**
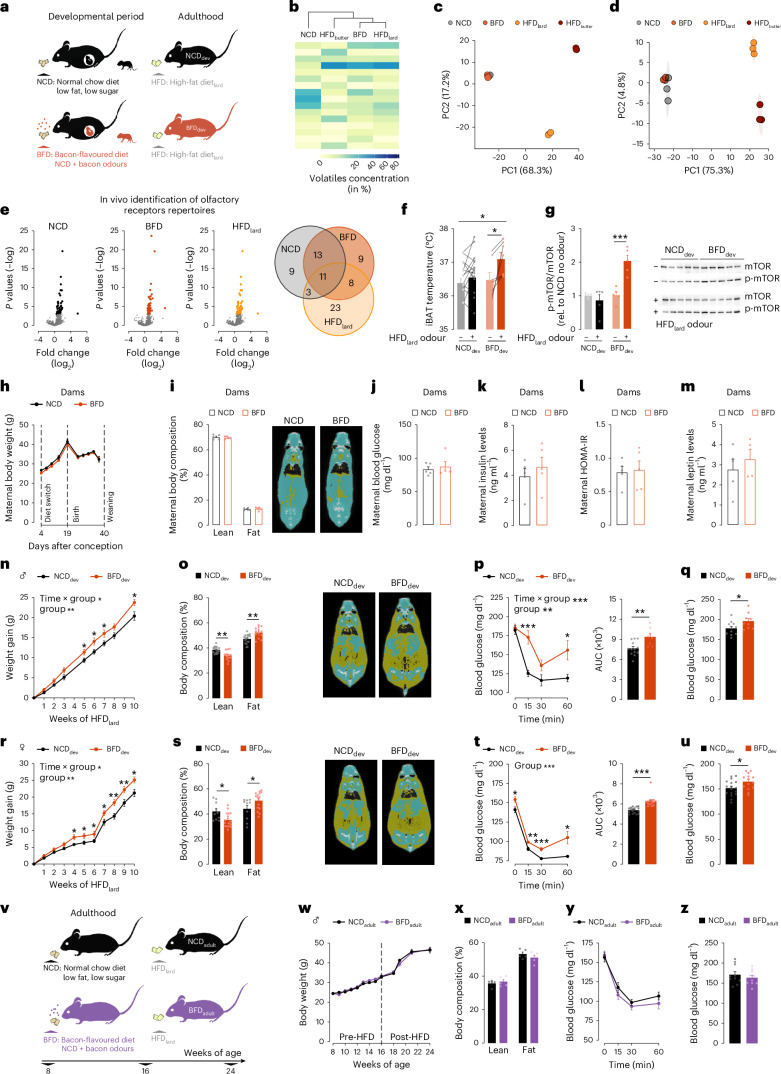
Developmental exposure to fat-related sensory cues is sufficient to exacerbate obesogenic responses to HFD_lard_. **a**, Paradigm of developmental exposure to fat-related sensory cues induced by feeding dams NCD (control group) or BFD. **b**, Hierarchical clustering of volatile profiles of NCD, BFD, butter-based HFD (HFD_butter_) and lard-based HFD (HFD_lard_). The colour scale represents the concentration of each volatile in per cent. Volatiles with a concentration >5% are depicted, volatiles <5% are shown in Extended Data Fig. 1. **c**,**d**, Principal-component analysis plots of the diets’ hydrophobic (lipids) (**c**) and diets’ polar (metabolites) components (**d**). **e**, In vivo identification of olfactory receptor repertoire of NCD, BFD and HFD_lard_ in adult mice. Volcano plot of olfactory receptor genes, fold change of odour-exposed (NCD, BFD and HFD_lard_) over no odour exposure using phospho-S6 ribosomal protein immunoprecipitation, with a Venn diagram of overlapping significant olfactory receptor genes (*n* = 5–6 per diet containing four main olfactory epithelia each; *P* < 0.001 considered significant following multiplicity adjustment). **f**, Interscapular brown adipose tissue (iBAT) temperature upon HFD_lard_ odour presentation in adult mice developmentally exposed to NCD (NCD_dev_) or BFD (BFD_dev_) naive to HFD_lard_ (two-way ANOVA; uncorrected Fisher’s LSD post hoc; *n* = 14,7; *P* diet = 0.0361, *P* BFD_*dev*_ NoOdour versus HFDodour = 0.0421). **g**, HFD_lard_ odour-induced changes in hepatic p-mTOR in fasted mice naive to HFD_lard_ (two-way ANOVA; Tukey’s post hoc; *n* = 5, 5; *P* = 0.0003). **h**–**m**, Metabolic phenotyping of dams fed either NCD or BFD. Maternal body weight across gestation and lactation (two-way repeated-measures ANOVA; *n* = 8, 9) (**h**). Maternal body composition and representative micro-CT image (two-tailed Wilcoxon rank-sum test; *n* = 5, 5) (**i**). Maternal fasted blood glucose (unpaired two-tailed *t*-test; *n* = 5, 5) (**j**). Maternal fasted insulin levels (unpaired two-tailed *t*-test; *n* = 5, 5) (**k**). Maternal insulin resistance defined by HOMA-IR (unpaired two-tailed *t*-test; *n* = 5, 5) (**l**). Maternal leptin levels (unpaired two-tailed *t*-test; *n* = 5, 4) (**m**). **n–u**, Metabolic phenotyping of NCD_dev_ or BFD_dev_ offspring fed HFD_lard_ during adulthood. Body weight gain of male offspring on HFD_lard_ (mixed-effects model; Tukey’s post hoc; *n* = 21, 20; *P* group = 0.006, *P* interaction = 0.022) (**n**). Body composition and representative micro-CT image of males (two-tailed Wilcoxon rank-sum test; *n* = 17, 13; *P* = 0.002) (**o**). Insulin tolerance test (ITT) and associated area under the curve (AUC) in males (time curve: two**-**way repeated-measures ANOVA; Tukey’s post hoc; *n* = 13, 8; *P* group = 0.0029, *P* interaction = 0.0003) (AUC: unpaired two-tailed *t*-test; *n* = 13, 8; *P* = 0.0032) (**p**). Male ad libitum resting blood glucose (unpaired two-tailed *t*-test; *n* = 11, 9; *P* = 0.0409) (**q**). Body weight gain of female offspring on HFD_lard_ (mixed-effects model; Tukey’s post hoc; *n* = 25, 29; *P* group = 0.0060, *P* interaction < 0.0001) (**r**). Body composition and representative micro-CT image of females (two-tailed Wilcoxon rank-sum test; *n* = 10,16; *P* = 0.029) (**s**). ITT and associated AUC in females (time curve: two-way repeated-measures ANOVA; Tukey’s post hoc; *n* = 17, 14; *P* group < 0.0001) (AUC: unpaired two-tailed *t*-test; *n* = 17, 14; *P* < 0.0001) (**t**). Female ad libitum resting blood glucose (unpaired two-tailed *t*-test; *n* = 17, 14; *P* = 0.0303) (**u**). **v**, Paradigm of adult exposure to NCD (NCD_adult_) or BFD (BFD_adult_) followed by HFD_lard_ feeding. **w**, Body weight curve (two-way repeated-measures ANOVA; *n* = 10, 10). **x**, Body composition (two-tailed Wilcoxon rank-sum test; *n* = 5, 5). **y**, ITT (time curve: two-way repeated-measures ANOVA; *n* = 10, 10). **z**, Resting ad libitum blood glucose (unpaired two-tailed *t*-test; *n* = 10,10) of NCD_adult_ and BFD_adult_. Statistics are depicted as for two-way repeated-measures ANOVA or mixed-effects model, group comparison and time × group interaction; Wilcoxon rank-sum test and *t*-test. Bars represent mean value. Error bars represent s.e.m. *P ≤ 0.05, **P ≤ 0.01, ***P ≤ 0.001. Source data

**Extended Data Fig. 1:**
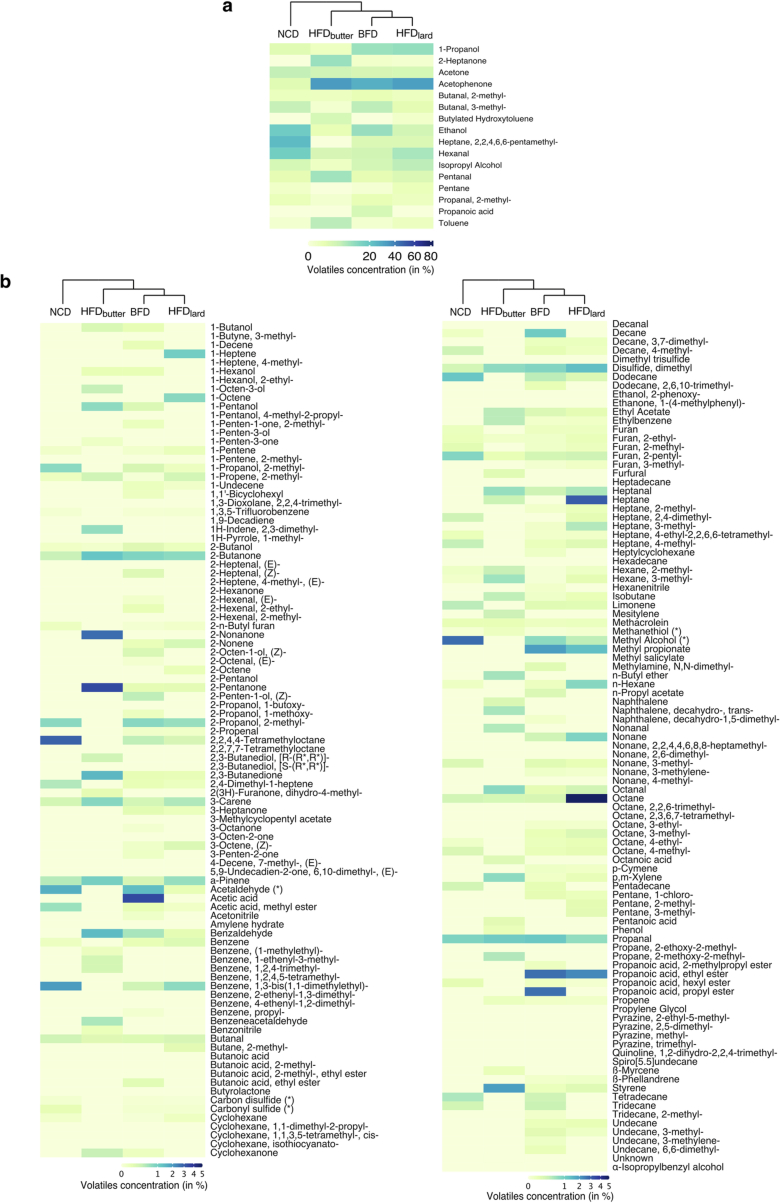
Diets’ volatile profiles. **(a-b)** Hierarchical clustering of the volatile profiles of normal chow diet (NCD), bacon-flavoured diet (BFD), butter-based high-fat diet (HFD_butter_) and lard-based HFD (HFD_lard_). The colour scale represents the concentration of each volatile in percent. Volatiles with a concentration **(a)** > 5% and **(b)** < 5% in at least one of the diets. Source data

**Extended Data Fig. 2:**
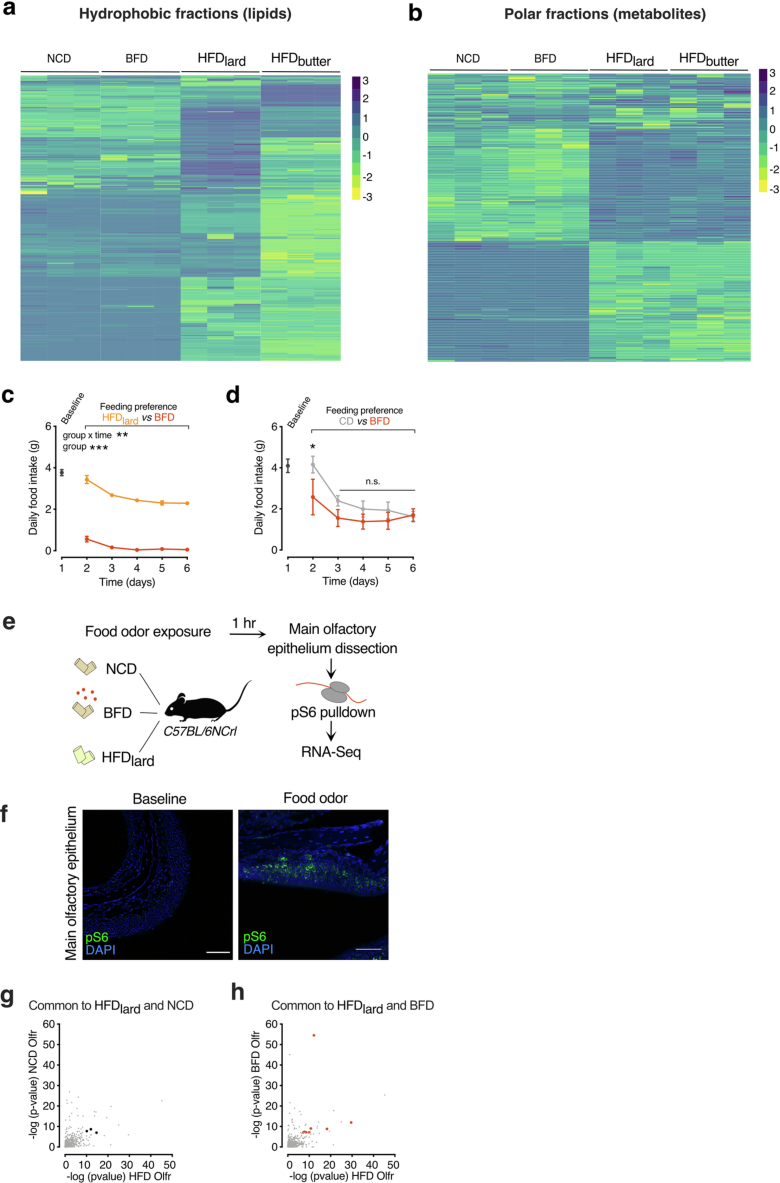
Characterization of the bacon-flavoured diet. (**a**-**b**) Diets’ hydrophobic and polar fractions (lipid and metabolite components). Heatmap of normal chow diet (NCD), bacon-flavoured diet (BFD), HFD_lard_ and HFD_butter_. (**a**) Lipidomic and (**b**) metabolomic profile. The colour scale represents the log-transformed, pareto scaled, TIC median normalized raw peak areas. **(c-d)** Two-choice diet preference test in adult C57BL/6NCrl exposed to **(c)** lard-based HFD (HFD_lard_) and bacon-flavoured diet (BFD) (two-way repeated measures ANOVA; uncorrected Fisher’s LSD post hoc; n = 8; *P* group < 0.0001; *P* interaction = 0.0015) and **(d)** control diet (CD) and BFD [two-way repeated measures ANOVA; uncorrected Fisher’s LSD post hoc; n = 7). **(e)** Paradigm of odour exposure and subsequent phosphorylated ribosome pulldown. **(f)** Example olfactory epithelium following overnight fasting and food odour exposure or baseline stained for pS6 (green) and DAPI (blue), scale bar = 50 μm. **(g-h)** Volcano plot of fold change of HFD_lard_-exposed olfactory receptors vs NCD or BFD. Highlighted olfactory receptors are significant for **(g)** only NCD and HFD_lard_ or **(h)** only BFD and HFD_lard_, but not the other food odour. [n = 5-6 per diet containing 4 main olfactory epithelia each; p-value ≤ 0.001 considered significant following multiplicity adjustment). Statistics are depicted as: for two-way repeated measures ANOVA, group comparison and time x group interaction. Bars represent mean value. Error bars represent SEM. * *P* ≤ 0.05, ** *P* ≤ 0.01, *** *P* ≤ 0.001. Source data

To dissociate the nutritive caloric components from the non-nutritive sensory components of HFD, we designed a diet that is isonutritional to commonly used normal chow diet (NCD) but flavoured with fat-related odours. To selectively mimic the commonly used lard-based HFD (HFD_lard_), we enriched NCD with bacon odours to create an isonutritional bacon-flavoured diet (BFD) (Fig. 1a). We first aimed to characterize the diet’s odour profile by performing a thermal-desorption gas chromatography–time-of-flight mass spectrometry (TD-GC–ToFMS)-based analysis of volatile organic compounds (VOCs) emitted by these diets. To investigate whether the BFD sensory profile resembles that of HFD_lard_ particularly, we also included a non-pork-based HFD: a butter fat-based HFD (HFD_butter_). These analyses revealed that the complex odour profiles of the diets comprised 155 volatiles, predominantly alcohols, aldehydes and ketones (Fig. 1b and Extended Data Fig. 1a,b). According to hierarchical cluster analysis, BFD and HFD_lard_ share high sensory similarities and their volatile profiles differ from those of HFD_butter_ and NCD (Fig. 1b and Extended Data Fig. 1a,b). To fully characterize BFD, we also performed an untargeted liquid chromatography-mass spectrometry (LC–MS)-based analysis of the hydrophobic (lipids) and polar fractions (metabolites) of BFD, NCD and the two HFDs (Fig. 1c,d, Extended Data Fig. 2a,b and Supplementary Tables 1 and 2). Principal-component analysis of the diets’ lipid (Fig. 1c) and polar profiles (Fig. 1d) unveiled that NCD and BFD overlap and are clearly distinct from HFD_lard_ and HFD_butter_. Hence, the sensory profile of BFD resembles more that of HFD_lard_, whereas its nutritional components are more similar to NCD. Consistent with the BFD’s low caloric and fat contents, mice subjected to a feeding preference test consumed almost exclusively palatable hypercaloric HFD_lard_ over BFD (Extended Data Fig. 2c). To ascertain that the low BFD consumption did not stem from any aversive or unpalatable properties, we also performed a feeding preference test between BFD and a novel NCD (control diet; CD) (Extended Data Fig. 2d). Although mice displayed a slight preference for CD over BFD the first day of exposure, no preference between the two diets was noticeable over time, hence, revealing that BFD feeding is not aversive to mice (Extended Data Fig. 2d).

To further corroborate the sensory similarities between BFD and HFD, we compared their ability to activate common olfactory receptors (Olfrs). We performed in vivo molecular profiling of olfactory receptors activated by NCD, BFD or HFD_lard_ odours using phosphorylated S6 ribosomal subunit capture (pS6-ribotrap)^30,31^ coupled to RNA sequencing (RNA-seq) in lean adult mice (Extended Data Fig. 2e). We confirmed that food odours induce phosphorylation of S6 in olfactory sensory neurons (OSNs) residing in the main olfactory epithelium (Extended Data Fig. 2f). NCD, BFD and HFD_lard_ odours activated 36, 41 and 45 olfactory receptors above the statistical threshold, respectively (Fig. 1e). Among the activated olfactory receptor repertoires of each diet, olfactory receptors activated by BFD showed a greater exclusive overlap with HFD_lard_ (eight common Olfrs) than NCD (three common Olfrs) (Fig. 1e and Extended Data Fig. 2g,h), suggesting that BFD shares an increased subjective olfactive similarity to HFD_lard_ compared with NCD.

**Fig. 2:**
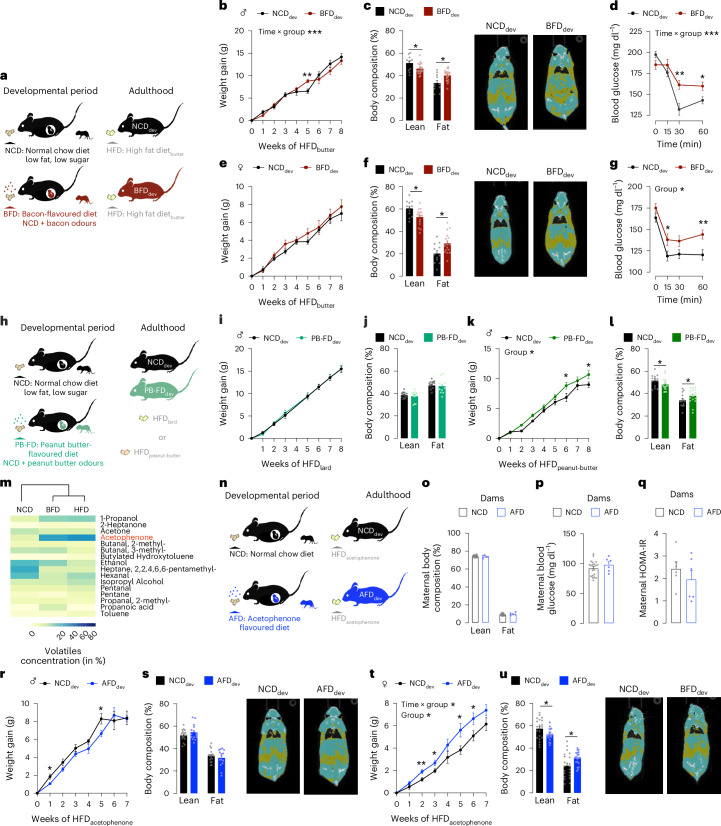
Developmental exposure to a single fat-related volatile is sufficient to prime obesity. **a**, Paradigm of developmental exposure to fat-related sensory cues induced by NCD or BFD feeding. Adult mice developmentally exposed to NCD (NCD_dev_) or BFD (BFD_dev_) are fed butter-based HFD (HFD_butter_) during adulthood. **b**–**g**, Metabolic phenotyping of NCD_dev_ and BFD_dev_ offspring on HFD_butter_. Body weight gain of male offspring on HFD_butter_ (mixed-effects model; Tukey’s post hoc; *n* = 11, 22; *P* interaction = 0.0006) (**b**). Body composition and representative micro-CT image of males (two-tailed Wilcoxon rank-sum test; *n* = 11, 22; *P* = 0.021) (**c**). ITT in males (two-way repeated-measures ANOVA; Tukey’s post hoc; *n* = 11, 22; *P* interaction < 0.0001) (**d**). Body weight gain of female offspring on HFD_butter_ (mixed-effects model; *n* = 12, 17) (**e**). Body composition and representative micro-CT image of females (two-tailed Wilcoxon rank-sum test; *n* = 12, 17; *P* = 0.028) (**f**). ITT in females (two-way repeated-measures ANOVA; Tukey’s post hoc; *n* = 12,17; *P* group = 0.0162) (**g**). **h**, Paradigm of developmental exposure to fat-related sensory cues induced by NCD or isonutritional PB-FD feeding in dams followed by either HFD_lard_ (**i**,**j**) or peanut butter-based HFD (HFD_peanut-butter_) (**k**,**l**) feeding in adulthood. **i**, Body weight gain of male offspring on HFD_lard_ (two-way repeated-measures ANOVA; *n* = 21, 13). **j**, Body composition of males on HFD_lard_ (two-tailed Wilcoxon rank-sum test; *n* = 17, 11). **k**, Body weight gain of male offspring on HFD_peanut-butter_ (mixed-effects model; Tukey’s post hoc; *n* = 16, 18; *P* group = 0.0397). **l**, Body composition of males on HFD_peanut-butter_ (two-tailed Wilcoxon rank-sum test; *n* = 14, 18; *P* = 0.042). **m**, Volatile profiles of NCD, BFD and HFD_lard_. The colour scale represents the concentration of each volatile in per cent. Volatiles with a concentration >5% are depicted, volatiles <5% are shown in Extended Data Fig. 1. **n**, Paradigm of developmental exposure induced by AFD feeding followed by acetophenone-flavoured HFD (HFD_acetophenone_) feeding in adulthood. **o**–**q**, Metabolic phenotyping of dams fed either NCD or AFD. Maternal body composition (two-tailed Wilcoxon rank-sum test; *n* = 20, 5) (**o**). Maternal blood fasting glycaemia (unpaired two-tailed *t*-test; *n* = 20, 6) (**p**). Maternal insulin resistance defined by HOMA-IR (unpaired two-tailed *t*-test; *n* = 6, 6) (**q**). **r**–**u**, Metabolic phenotyping of NCD_dev_ or AFD_dev_ mice fed HFD_acetophenone_ during adulthood. Body weight gain of male offspring on HFD_acetophenone_ (mixed-effects model; *n* = 19,19) (**r**). Body composition and representative micro-CT image of males (two-tailed Wilcoxon rank-sum test; *n* = 18,15) (**s**). Body weight gain of female offspring on HFD_acetophenone_ (mixed-effects model; Tukey’s post hoc; *n* = 22,17; *P* group = 0.0168; *P* interaction = 0.0126) (**t**). Body composition and representative micro-CT image of females (two-tailed Wilcoxon rank-sum test; *n* = 21,20; *P* = 0.046) (**u**). Statistics are depicted as for two-way repeated-measures ANOVA or mixed-effects model, group comparison and time × group interaction; Wilcoxon rank-sum test and *t*-test. Bars represent mean value. Error bars represent s.e.m. *P ≤ 0.05, **P ≤ 0.01, ***P ≤ 0.001. Source data

**Extended Data Fig. 3:**
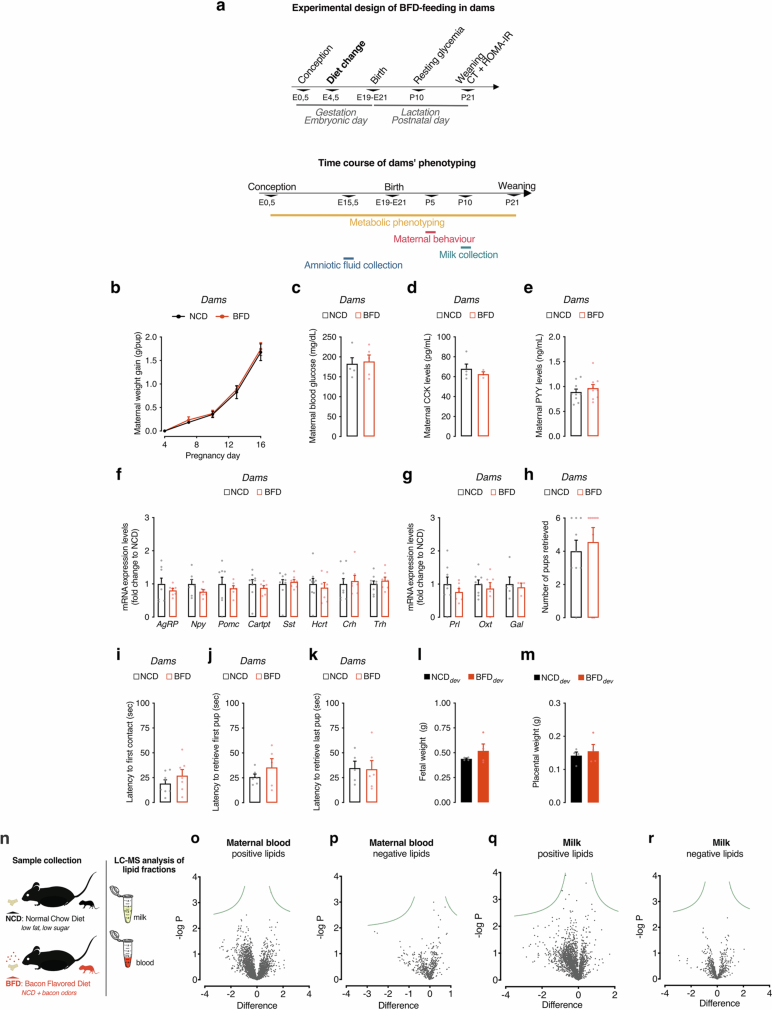
Maternal BFD feeding does not alter maternal health and behaviour, nor blood and milk lipid profiles. **(a)** Overview of the timeline and different cohorts performed to phenotype dams fed normal chow diet (NCD) or bacon-flavoured diet (BFD). **(b)** Weight gain per pup through gestation [two-way repeated measures ANOVA; n = 5,6). **(c)** Maternal ad libitum resting blood glucose [unpaired two-tailed t-test; n = 5,5). **(d)** Maternal cholecystokinin (CCK) levels [unpaired two-tailed t-test; n = 5,3). **(e)** Maternal peptide YY (PYY) levels [unpaired two-tailed t-test; n = 10,10). **(f-g)** Hypothalamic gene expression of **(f)** energy homeostasis-related genes and **(g)** maternal behaviour-related genes [multiple unpaired two-tailed t-test; n = 9,7;8,7). **(h-k)** Pup retrieval test [unpaired two-tailed t-test]. **(h)** Total number of pups retrieved [n = 9,9]. **(i)** Latency to first contact [n = 8,7]. **(j-k)** Retrieval latencies to retrieve **(j)** the first [n = 6,5] and **(k)** the last pup [n = 5,6]. **(l)** Fetal [unpaired two-tailed t-test; n = 4,4] and **(m)** placental weight [unpaired two-tailed t-test; n = 4,4] at embryonic day 15.5 of offspring exposed to NCD (NCD_*dev*_) and BFD **(**BFD_*dev*_) through maternal diet. **(n)** Paradigm for milk and blood collection in dams fed normal chow diet (NCD) or bacon-flavoured diet (BFD). **(o-r)** Volcano plot for the lipidomic profiling of: **(o-p)** maternal blood and **(q-r)** milk. Green lines indicate multiplicity adjusted p = 0.05. Bars represent mean value. Error bars represent SEM. Source data

After thoroughly characterizing the BFD, we next used this diet to generate a model of developmental exposure to fat-related sensory cues. At gestational day 4.5, dams were either maintained on NCD or switched to BFD until weaning of the pups to mimic sensory experiences associated with HFD_lard_ feeding during development without its nutritive consequences (Fig. 1a and Extended Data Fig. 3a). To characterize our model, we aimed to validate that exposure to BFD could indeed mimic the exposure to HFD_lard_. Under normal conditions, mice naive to a palatable diet (such as HFDs) show minimal responses to its sensory signals at the first encounter, and the sensory response is contingent and tuned by its consumption^20^. Therefore, we sought to test whether developmental exposure to BFD is sufficient to prime a differential response to HFD_lard_ odour in adult offspring naive to HFD_lard_. We measured two sensory-evoked metabolic readouts: (1) HFD_lard_ odour-induced increase in interscapular brown adipose tissue (iBAT) temperature^21^ (Fig. 1f) and (2) priming of hepatic post-prandial responses, for example mTOR phosphorylation (p-mTOR)^22^ (Fig. 1g). In control mice exposed to NCD during development (NCD_dev_) and naive to HFD_lard_, we found that HFD_lard_ odour exposure did not induce changes in iBAT temperature or hepatic p-mTOR responses (Fig. 1f,g). In contrast, iBAT temperature and hepatic p-mTOR increased upon HFD_lard_ odour exposure in mice developmentally exposed to BFD (BFD_dev_) but naive to HFD_lard_ (Fig. 1f,g). Thus, developmental exposure to BFD is sufficient to emulate sensory exposure to HFD_lard_ and to prime metabolic responses to HFD_lard_ before its first encounter.

To further characterize our mouse model of developmental exposure to fat-related sensory cues, we conducted a thorough analysis of the dams’ metabolism and behaviour. BFD feeding did not alter maternal body weight (Fig. 1h), weight gain per pup (Extended Data Fig. 3b) or body composition (Fig. 1i). Additionally, fasting (Fig. 1j) and ad libitum-fed glycaemia (Extended Data Fig. 3c), circulating insulin levels (Fig. 1k) and insulin resistance (Fig. 1l) were comparable between BFD-fed and control dams. Circulating levels of the hunger and satiety hormones leptin (Fig. 1m), CCK (Extended Data Fig. 3d) and PYY (Extended Data Fig. 3e) were also similar between the two groups. Furthermore, BFD feeding had no impact on the expression of key hypothalamic peptides involved in regulating feeding and energy balance (Extended Data Fig. 3f). Hypothalamic-pituitary-adrenal and hypothalamic-pituitary-thyroid axes were also unaffected (Extended Data Fig. 3f). Maternal behaviour remained unchanged between BFD- and NCD-fed dams as illustrated by similar expression of pivotal hormones governing parental behaviour (prolactin, oxytocin and galanin; Extended Data Fig. 3g) and similar performance in a pup retrieval test (Extended Data Fig. 3h–k). Moreover, BFD feeding did not alter fetal and placental weights (Extended Data Fig. 3l,m), further demonstrating that maternal health and fetal development were unaffected.

As maternal lipid profiles are well-known contributors to metabolic programming^32,33^, we also performed untargeted LC–MS analyses of extracted lipid fractions on blood and milk of dams fed NCD or BFD (Extended Data Fig. 3n). These analyses revealed that BFD feeding in dams did not significantly alter blood or milk lipid composition (Extended Data Fig. 3o–r). Hence, we successfully developed a model for developmental exposure to fat-related sensory cues independent of maternal insulin resistance, weight gain, adiposity and changes in maternal lipid components.

## Exacerbated obesogenic response in sensory programmed offspring

**Extended Data Fig. 4:**
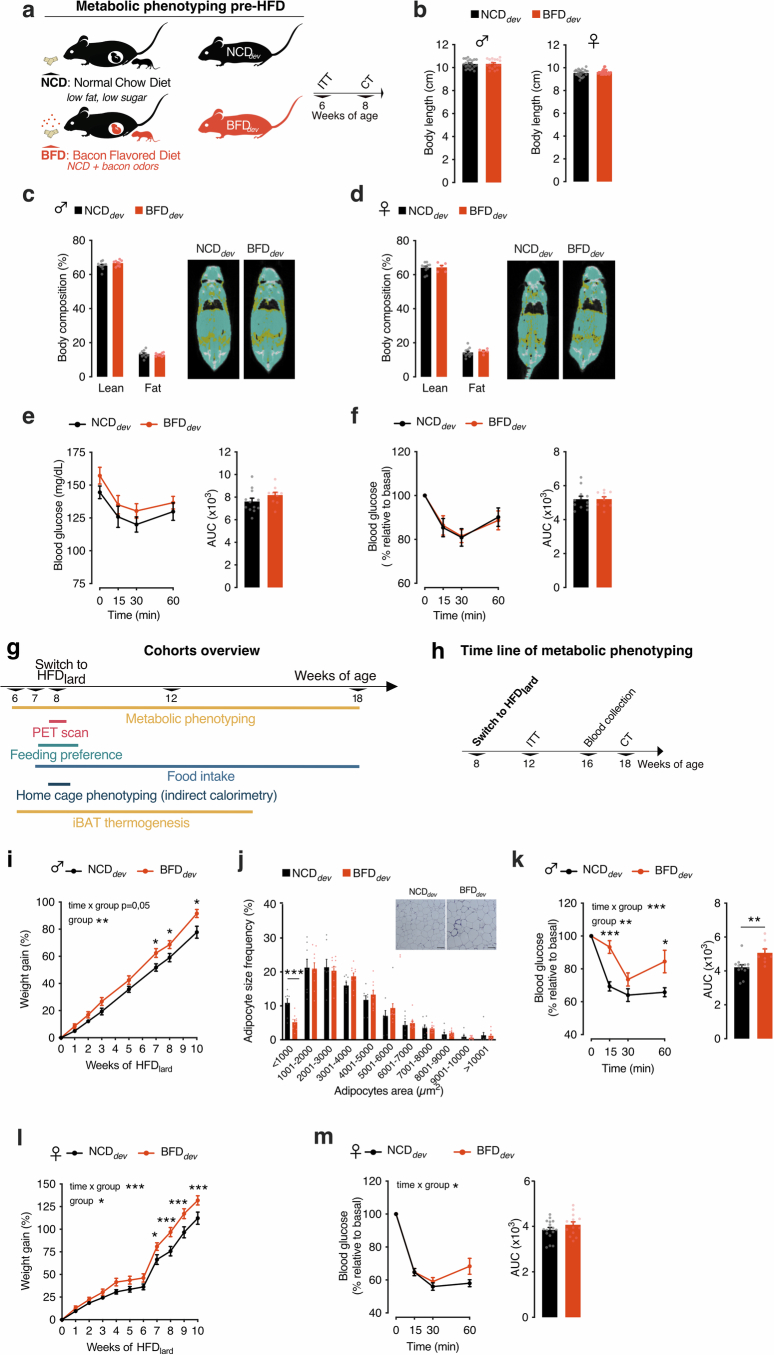
Developmental exposure to fat-related sensory cues does not alter adiposity and insulin sensitivity prior to HFD_lard_ feeding. **(a)** Paradigm for the metabolic phenotyping before the switch to lard-based high-fat diet (HFD_lard_) of offspring developmentally exposed to normal chow diet (NCD_*dev*_) or bacon-flavoured diet (BFD_*dev*_). **(b)** Body length of males [unpaired two-tailed t-test; n = 22,19] and females [unpaired two-tailed t-test; n = 25,29]. **(c-d)** Body composition and representative micro-CT images pre-HFD_lard_ of **(c)** males [two-tailed Wilcoxon rank-sum test; n = 10,9] and **(d)** females [two-tailed Wilcoxon rank-sum test; n = 11,5]. **(e-f)** Insulin tolerance test (ITT) and associated area under the curve (AUC) pre-HFD_lard_ males: **(e)** absolute glucose levels [time curve: two-way repeated measures ANOVA; n = 12,10] [AUC: unpaired two-tailed t-test; n = 12,10] and **(f)** relative to baseline [time curve: two-way repeated measures ANOVA; n = 12,10] [AUC: unpaired two-tailed t-test; n = 12,10]. **(g)** Overview of the timeline and different cohorts performed to phenotype NCD_*dev*_ and BFD_*dev*_ mice. **(h)** Timeline for post-HFD_lard_ metabolic phenotyping of NCD_*dev*_ or BFD_*dev*_ mice. **(i)** Body weight gain of male offspring on HFD_lard_ in percentage [two-way repeated measures ANOVA; Tukey’s post hoc; n = 21,20; *P* group = 0.0029; *P* interaction = 0.0509]. **(j)** Size distribution of adipocytes in perigonadal white adipose tissue [two-way repeated measures ANOVA; Sidak’s post hoc; n = 8,10; *P* < *1000* = 0.0006]. **(k)** Insulin tolerance test (ITT) and associated area under the curve (AUC) relative to baseline after 4 weeks of HFD_lard_ in males [time curve: two-way repeated measures ANOVA; Tukey’s post hoc; n = 13,8; *P* group = 0.0024; *P* interaction = 0.0002] [AUC: unpaired two-tailed t-test, n = 13,8; *P* = 0.0044]. **(l)** Body weight gain of female offspring on HFD_lard_ in percentage [two-way repeated measures ANOVA; Tukey’s post hoc; n = 25,27; *P* group = 0.0153; *P* interaction = 0.0002]. **(m)** ITT and associated AUC in females [time curve: two-way repeated measures ANOVA; n = 17,14; *P* interaction = 0.0375] [AUC: unpaired two-tailed t-test; n = 17,14]. Statistics are depicted as: for two-way repeated measures ANOVA or mixed-effects model, group comparison and time x group interaction. Bars represent mean value. Error bars represent SEM. * p ≤ 0.05, ** p ≤ 0.01, *** p ≤ 0.001. Source data

To test whether exposure to HFD_lard_-related sensory cues during development alters the response to HFD_lard_ feeding in adulthood, we next assessed whether early exposure to fat-related sensory cues impacts the onset of diet-induced obesity and associated metabolic alterations. Before the switch to HFD_lard_, body length (Extended Data Fig. 4a,b), body composition (Extended Data Fig. 4c,d), and insulin sensitivity (Extended Data Fig. 4e,f) were identical between BFD_dev_ and control NCD_dev_ animals. To determine the long-term influence of developmental exposure to BFD on metabolic health, NCD_dev_ and BFD_dev_ offspring were switched to HFD_lard_ at 8 weeks of age (Extended Data Fig. 4g,h). HFD_lard_ feeding induced an aggravated body weight gain (Fig. 1n and Extended Data Fig. 4i) and adiposity (Fig. 1o and Extended Data Fig. 4j) in male BFD_dev_ animals compared with control NCD_dev_. Similarly, insulin resistance (Fig. 1p and Extended Data Fig. 4k) and higher resting glycaemia (Fig. 1q) induced by HFD_lard_ feeding were increased in BFD_dev_ mice compared with NCD_dev_. Female BFD_dev_ mice also displayed exacerbated HFD-induced weight gain (Fig. 1r and Extended Data Fig. 4l), adiposity (Fig. 1s), insulin resistance (Fig. 1t and Extended Data Fig. 4m) and hyperglycaemia (Fig. 1u). Hence, developmental exposure to BFD exacerbates the obesogenic responses to HFD_lard_ to a comparable extent in both sexes, demonstrating that fat-related sensory cues during early life exert potent and generalized metabolic defects in adulthood.

**Extended Data Fig. 5:**
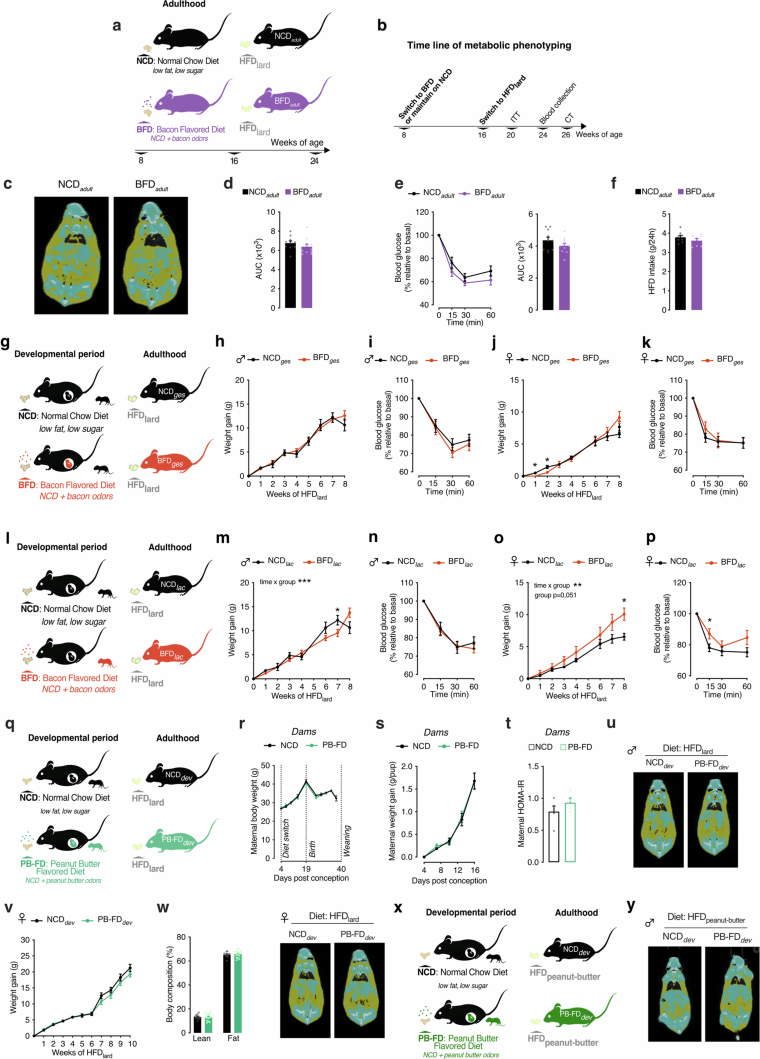
Exposure to fat-related sensory cues during development, but not during adulthood, influences diet-induced obesity. **(a)** Paradigm of adult exposure to NCD (NCD_*adult*_) or BFD (BFD_*adult*_) followed by lard-based high-fat diet (HFD_lard_) feeding. **(b)** Overview of the metabolic phenotyping time line. **(c)** Representative micro-CT image (related to Fig. 1x) after 8 weeks of HFD_lard_. **(d)** Area under the curve (AUC) of insulin tolerance test (ITT) in absolute glucose levels (related to Fig. 1y) after 4 weeks of HFD_lard_ [AUC: unpaired two-tailed t-test; n = 10,10]. **(e)** Insulin tolerance test (ITT) and associated AUC after 4 weeks of HFD_lard_ relative to baseline [time curve: two-way repeated measures ANOVA, n = 10,10] [AUC: unpaired two-tailed t-test; n = 10,10]. **(f)** Daily HFD_lard_ intake consumed during ad libitum access [unpaired two-tailed t-test; n = 8,7]. **(g)** Paradigm of developmental exposure to NCD and BFD during gestation exclusively (NCD_*ges*_ and BFD_*ges*_) followed by HFD_lard_ feeding in adulthood. **(h)** Body weight gain of male offspring on HFD_lard_ [mixed-effects model; n = 15,13]. **(i)** ITT of male offspring on HFD_lard_ [two-way repeated measures ANOVA; n = 22,16]. **(j)** Body weight gain of female offspring on HFD_lard_ [mixed-effects model; Tukey’s post hoc; n = 18,10]. **(k)** ITT of female offspring on HFD_lard_ [two-way repeated measures ANOVA; n = 30,15]. **(l)** Paradigm of developmental exposure to NCD and BFD during lactation exclusively (NCD_*lac*_ and BFD_*lac*_) followed by HFD_lard_ feeding in adulthood. **(m)** Body weight gain of male offspring on HFD_lard_ [mixed-effects model; Tukey’s post hoc; n = 15,20; *P* interaction = 0.0009]. **(n)** ITT of male offspring on HFD_lard_ [two-way repeated measures ANOVA; n = 22,24]. **(o)** Body weight gain of female offspring on HFD_lard_ [mixed-effects model; Tukey’s post hoc; n = 18,10; *P* group = 0.0512; *P* interaction = 0.0026]. **(p)** ITT of female offspring on HFD_lard_ [two-way repeated measures ANOVA; Tukey’s post hoc; n = 30,12]. **(q)** Paradigm of developmental exposure to fat-related sensory cues induced by normal chow diet (NCD) or isonutritional peanut butter-flavoured diet (PB-FD) feeding. **(r-u)** Metabolic phenotyping of dams fed either NCD or PB-FD. **(r)** Maternal weight across gestation and lactation [two-way repeated measures ANOVA; n = 8,7]. **(s)** Weight gain per pup through gestation of dams fed NCD or PB-FD [two-way repeated measures ANOVA; n = 5,4]. **(t)** Maternal insulin resistance defined by HOMA-IR [unpaired two-tailed t-test; n = 5,4]. **(u-y)** Metabolic phenotyping of offspring on HFD_lard_ exposed to NCD_*dev*_ or PB-FD_*dev*_ **(u)** Representative micro-CT image of HFD_lard_-fed males (related to Fig. 2j). **(v)** Body weight gain of female offspring on HFD_lard_ [two-way repeated measures ANOVA; n = 25,20]. **(w)** Body composition and representative micro-CT image of HFD_lard_-fed females [two-tailed Wilcoxon rank-sum test; n = 10,14]. **(x)** Paradigm of developmental exposure to fat-related sensory cues induced by NCD or PB-FD feeding followed by peanut butter-based high-fat diet (HFD_peanut-butter_) feeding in adulthood. **(y)** Representative micro-CT image of HFD_peanut-butter_-fed males (related to Fig. 2l). Statistics are depicted as: for two-way ANOVA or mixed-effects model, group comparison and time x group interaction. Bars represent mean value. Error bars represent SEM. * p ≤ 0.05, *** p ≤ 0.001. Source data

To assess whether the adverse metabolic outcomes triggered by BFD are selectively associated with the exposure during development, we exposed animals to BFD exclusively during adulthood. Adult male mice were fed BFD (BFD_adult_) or NCD (NCD_adult_) for 8 weeks and then fed HFD_lard_ to assess the influence of BFD feeding on obesity development (Fig. 1v and Extended Data Fig. 5a–f). BFD feeding exclusively during adulthood (BFD_adult_) had no consequences on HFD_lard_-induced weight gain (Fig. 1w), adiposity (Fig. 1x and Extended Data Fig. 5c), insulin resistance (Fig. 1y and Extended Data Fig. 5d,e), or hyperglycaemia (Fig. 1z). The exacerbated obesogenic responses in BFD_dev_ animals are, therefore, triggered only by exposure to BFD during development. Thus, early life is a critical period of vulnerability for the metabolic programming induced by fat-related sensory cues, and developmental exposure to fat-related sensory cues is sufficient to exacerbate HFD_lard_-induced obesity, independent of maternal obesity or insulin resistance.

To investigate whether the sensory programming of metabolism is triggered by a specific developmental period, we selectively fed dams with BFD during gestation (Extended Data Fig. 5g–k) or lactation (Extended Data Fig. 5l–p). Exposure to BFD during gestation (BFD_ges_) alone did not alter HFD_lard_-induced obesity in either males (Extended Data Fig. 5h,i) or females (Extended Data Fig. 5j,k). However, while BFD exposure during lactation (BFD_lac_) did not have a clear effect on obesity development in males (Extended Data Fig. 5m,n), it significantly increased weight gain (Extended Data Fig. 5o) and decreased insulin sensitivity (Extended Data Fig. 5p) in females. Collectively these data suggest that lactation might be a period of highest sensitivity to fat sensory cues in females, while exposure to BFD throughout the entire developmental period is required for the exacerbated responses to HFD_lard_ in males.

## The sensory programming of metabolism generalizes to non-pork-based HFD and other fat-related odours

Having established that developmental exposure to BFD exacerbates obesity in response to HFD_lard_, we next aimed to determine whether these metabolic responses were specific to the pork-based HFD that shares high sensory similarities with the BFD. We therefore exposed 8-week-old BFD_dev_ and control NCD_dev_ animals to a butter fat-based HFD (HFD_butter_) (Fig. 2a). While HFD_butter_ only had a minor influence on body weight gain (Fig. 2b) in male BFD_dev_ animals compared with control NCD_dev_ animals, it increased their adiposity (Fig. 2c), a primary determinant of metabolic and cardiovascular risks^34,35^. HFD_butter_ also decreased insulin sensitivity in BFD_dev_ males (Fig. 2d). A similar phenotype was observed in female BFD_dev_ mice with unchanged body weight gain (Fig. 2e), increased adiposity (Fig. 2f) and insulin resistance (Fig. 2g). Altogether, these results uncover that exposure to fat sensory cues mimicking HFD_lard_ also primes obesity in response to other HFDs, revealing that the sensory programming of metabolism can generalize to other hypercaloric diets.

After demonstrating that the heightened obesogenic responses induced by developmental exposure to bacon sensory cues apply to other HFDs, we next investigated whether non-nutritive sensory programming can be generalized to other fat-related sensory cues. Therefore, we performed an identical developmental paradigm using a peanut butter-flavoured diet (PB-FD) as a separate fat-related odour (Fig. 2h). PB-FD feeding did not alter maternal body weight (Extended Data Fig. 5q,r), weight gain per pup (Extended Data Fig. 5s) nor insulin sensitivity (Extended Data Fig. 5t). We first tested the responses of PB-FD_dev_ and NCD_dev_ animals to HFD_lard_. Animals developmentally exposed to PB-FD (PB-FD_dev_) showed similar obesogenic responses to HFD_lard_ feeding compared with NCD_dev_ with indistinguishable weight gain (Fig. 2i) and adiposity (Fig. 2j and Extended Data Fig. 5u) in males and females (Extended Data Fig. 5v,w). Hence, developmental exposure to PB-related odours does not exacerbate the obesogenic responses to HFD_lard_. Of note, these results further demonstrate that the metabolic phenotype observed in BFD_dev_ animals is not triggered by a generalized effect of increased sensory enrichment or potential maternal stress^36,37^.

To explore whether the absence of exacerbated obesogenic responses could originate from the type of HFD used (lard versus peanut butter), we assessed the responses of PB-FD_dev_ and NCD_dev_ control animals to a peanut butter-based HFD (HFD_peanut-butter_) (Extended Data Fig. 5x). HFD_peanut-butter_ feeding increased weight gain (Fig. 2k) and adiposity (Fig. 2l and Extended Data Fig. 5y) in PB-FD_dev_ mice compared with NCD_dev_ controls. Overall, these experiments demonstrate that the sensory programming of metabolism by fat-related sensory cues extends to other diets. Moreover, these findings suggest that, although identical sensory profiles between the developmental diet and the obesogenic diets encountered in adulthood are not required for metabolic effects, a certain degree of similarity may be necessary.

## Developmental exposure to a single HFD-enriched volatile is sufficient to prime obesity

**Extended Data Fig. 6:**
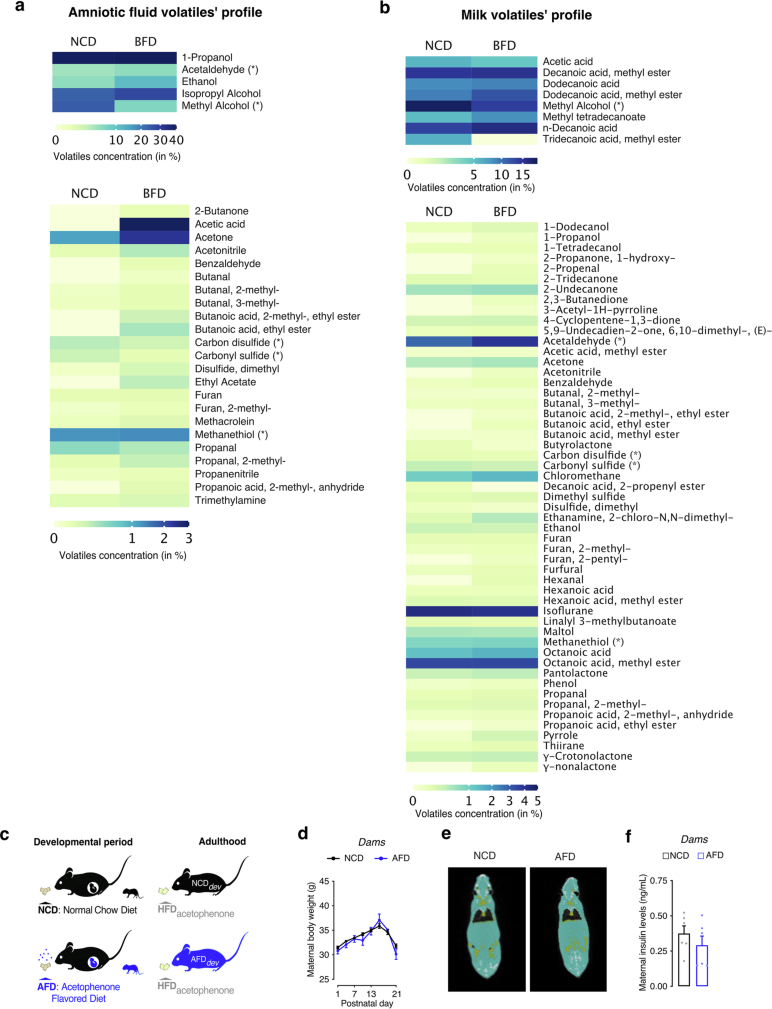
BFD feeding in dams changes amniotic fluid and milk volatile profiles. **(a-b)** Volatile profiles of **(a)** amniotic fluid and **(b)** milk of dams fed normal chow diet (NCD) or bacon-flavoured diet (BFD). The colour scale represents the concentration of each volatile in percentage. Top and bottom heatmaps depict volatiles >5% and <5%, respectively. **(c)** Paradigm of developmental exposure to acetophenone-flavoured diet (AFD) feeding followed by acetophenone-flavoured HFD (HFD_acetophenone_) feeding in adulthood. **(d)** Maternal weight during lactation [mixed-effects model; n = 20,6]. **(e)** Representative micro-CT image (related to Fig. 2o). **(f)** Maternal fasted insulin levels [unpaired two-tailed t-test; n = 6,6]. Bars represent mean value. Error bars represent SEM. Source data

To further confirm the influence of HFD sensory cues on long-term metabolic health, we investigated whether early exposure to a single fat-related volatile could affect metabolic health. Volatile analyses revealed several molecules enriched in BFD and HFD, including acetophenone, 1-propanol, benzaldehyde, heptanal, 2-pentanone and ethyl acetate, which are known to contribute to the specific odour of bacon^38^ (Fig. 2m and Extended Data Fig. 1). Given that volatile compounds present in the maternal diet can be transferred to the amniotic fluid and milk, we also compared the volatile profiling of those fluids between BFD and NCD dams (Extended Data Fig. 6a,b), revealing clear differences between the two groups. Specifically, these analyses revealed an increase in BFD- and HFD-enriched volatiles, such as benzaldehyde and 1-propanol (in milk), and volatiles potentially derived from degradation of food-derived volatiles, such as acetone, one of the degradation products of acetophenone (Extended Data Fig. 6a,b). Collectively, these results highlight that BFD feeding influences the volatile profile of amniotic fluid and milk and subsequently modifies the in utero and neonatal offspring’s food sensory experiences.

We then tested whether developmental exposure to a single HFD-related volatile could influence obesity susceptibility. Among the several volatiles enriched in BFD and HFD_lard_, we focused on acetophenone as a proof of principle, as it is the most abundant volatile in both diets (Fig. 2m). To model developmental exposure to acetophenone odours, dams were fed NCD flavoured with acetophenone (acetophenone-flavoured diet; AFD) during gestation and lactation (Fig. 2n). AFD feeding did not affect maternal weight gain (Extended Data Fig. 6c,d), adiposity (Fig. 2o and Extended Data Fig. 6e), glycaemia (Fig. 2p), insulin resistance (Fig. 2q) nor insulin levels (Extended Data Fig. 6f). At 8 weeks of age, offspring of AFD-fed (AFD_dev_) and control (NCD_dev_) dams were switched to HFD_lard_ flavoured with acetophenone (HFD_acetophenone_) (Fig. 2n). Weight gain (Fig. 2r) and adiposity (Fig. 2s) induced by HFD_acetophenone_ were similar in AFD_dev_ and NCD_dev_ males. In contrast, AFD_dev_ females exhibited increased weight gain (Fig. 2t) and adiposity (Fig. 2u) in response to HFD_acetophenone_ compared with NCD_dev_ controls (interaction sex × developmental diet × time: *P* = 0.005). These findings further underscore the critical role of food sensory cues in the developmental origin of obesity, demonstrating that exposure to a single fat-related sensory volatile during development is sufficient to exacerbate obesogenic responses in a sexually dimorphic manner.

## Developmental exposure to fat-related odours alters dopamine response to food cues and primes feeding preference

**Fig. 3:**
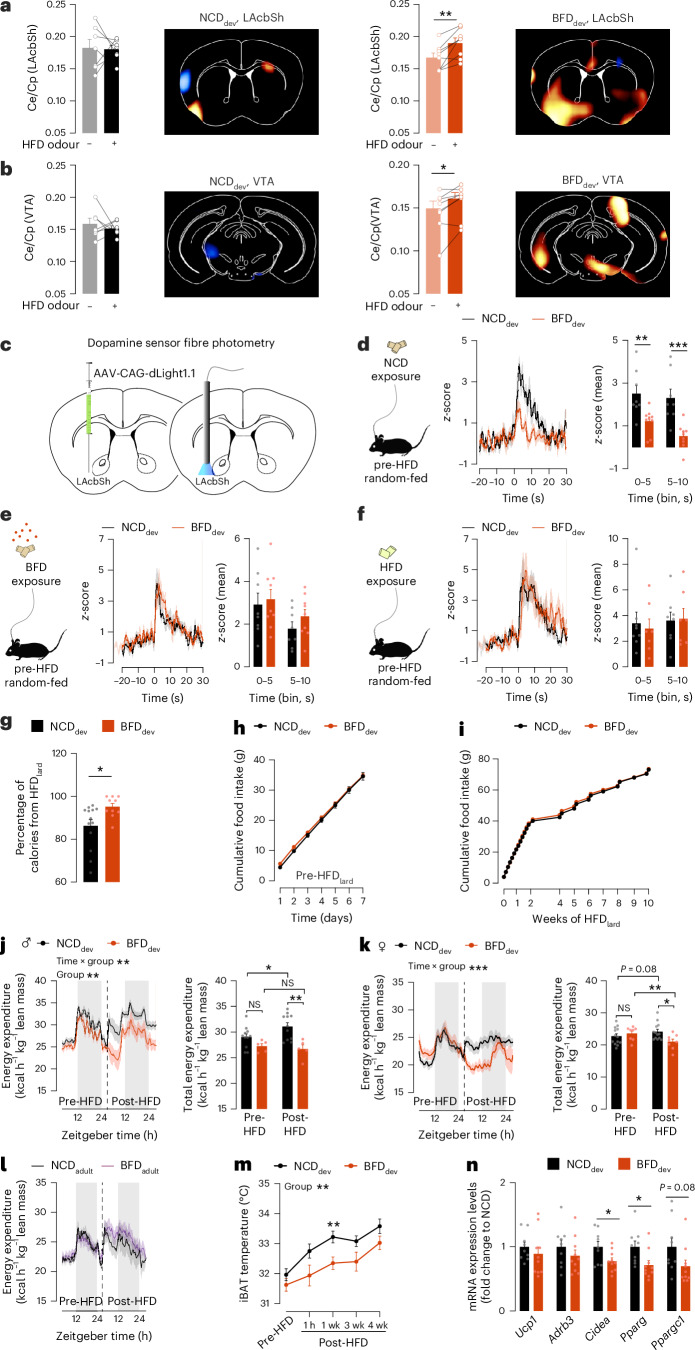
Developmental exposure to fat-related odours alters dopaminergic response, gates preference towards HFD_lard_ and decreases energy expenditure. **a**,**b**, Lard-based HFD (HFD_lard_) odour-induced differential glucose transport based on fluorodeoxyglucose (^18^FDG) PET kinetics in adult mice exposed to NCD (NCD_dev_) or BFD (BFD_dev_) during development pre-HFD_lard_ in the lateral nucleus accumbens shell (LAcbSh) (paired two-tailed *t*-test; *n* = 7, 8; *P* = 0.0073) (**a**) and ventral tegmental area (VTA) (paired two-tailed *t*-test; *n* = 7, 8; *P* = 0.0213) (**b**). The colour code on the representative PET images depicts the paired *t*-test value for each voxel. Relative increases in glucose metabolism upon HFD_lard_ odour exposure are colour-coded in yellow to orange, whereas decreases are colour-coded in blue. **c**, Schematic of unilateral viral delivery of the dopamine optical sensor dLight1.1 in the LAcbSh, cannula implantation for fibre photometry analysis of dopamine release, and food exposure paradigm used for dLight1.1 recordings. **d**–**f**, Dopamine response to food in adult NCD_dev_ and BFD_dev_ mice pre-HFD_lard_. Dopamine sensor fluorescence depicted as *z*-score and 5-s binned average upon approach to a NCD pellet (two-way ANOVA; *n* = 8, 9; uncorrected Fisher’s LSD post hoc; *P* group < 0.0001; *P* time_0–5_ = 0.0055; *P* time_5–10_ = 0.0003) (**d**), a BFD pellet (two-way ANOVA; *n* = 8, 9) (**e**) or a HFD_lard_ pellet (two-way ANOVA; *n* = 8, 7) (**f**). **g**, Percentage of calories from HFD_lard_ during the first day of a two-choice diet preference test (unpaired two-tailed *t*-test; *n* = 13, 10; *P* = 0.0206). **h**, Cumulative food intake of NCD_dev_ and BFD_dev_ mice before the switch to HFD_lard_ (two-way repeated-measures ANOVA; *n* = 8, 8). **i**, Cumulative food intake of HFD_lard_ during 10 weeks on HFD_lard_ (two-way repeated-measures ANOVA; *n* = 8, 8). **j**,**k**, Energy expenditure of NCD_dev_ and BFD_dev_ mice pre-and post-HFD_lard_ feeding and total EE in: males (time curve: two-way repeated-measures ANOVA; *n* = 11, 5; *P* group = 0.0054; *P* interaction = 0.0023) (total EE, linear mixed-effects models; Holm–Bonferroni post hoc; *n* = 11, 5; *P* interaction = 0.061; *P* post-HFD NCD_dev_ versus post-HFD BFD_dev_ = 0.004, *P* pre-HFD versus post-HFD NCD_dev_ = 0.049) (**j**), females (time curve: two-way repeated-measures ANOVA; *n* = 13, 8; *P* interaction < 0.0001) (total EE, linear mixed-effects models; Holm–Bonferroni post hoc; *n* = 13, 8; *P* interaction < 0.001; *P* post-HFD NCD_dev_ post-HFD BFD_dev_ = 0.048; *P* pre-HFD post-HFD BFD_dev_ = 0.009; *P* pre-HFD post-HFD NCD_dev_ = 0.08) (**k**). NS, not significant. **l**, Energy expenditure of mice exposed in adulthood to NCD (NCD_adult_) or BFD (BFD_adult_) pre-and post-HFD_lard_ feeding (two-way repeated-measures ANOVA; *n* = 7, 8). **m**, Interscapular brown adipose tissue (iBAT) temperature recordings in NCD_dev_ and BFD_dev_ mice pre-and post-HFD_lard_ feeding (two-way repeated-measures ANOVA; Tukey’s post hoc; *n* = 10,8; *P* group = 0.0047). **n**, Gene expression of thermogenesis-related genes in iBAT in NCD_dev_ and BFD_dev_ mice fed HFD_lard_ for 1 week (multiple unpaired two-tailed *t*-test; *n* = 9, 10; *P* *Cidea* = 0.034; *P Pparg* = 0.022). Statistics are depicted as: for two-way repeated-measures ANOVA or mixed-effects model, group comparison and time × group interaction; for multiple unpaired *t*-test and *t*-test. Bars represent mean value. Error bars represent s.e.m. *P ≤ 0.05, **P ≤ 0.01, ***P ≤ 0.001. Source data

**Extended Data Fig. 7:**
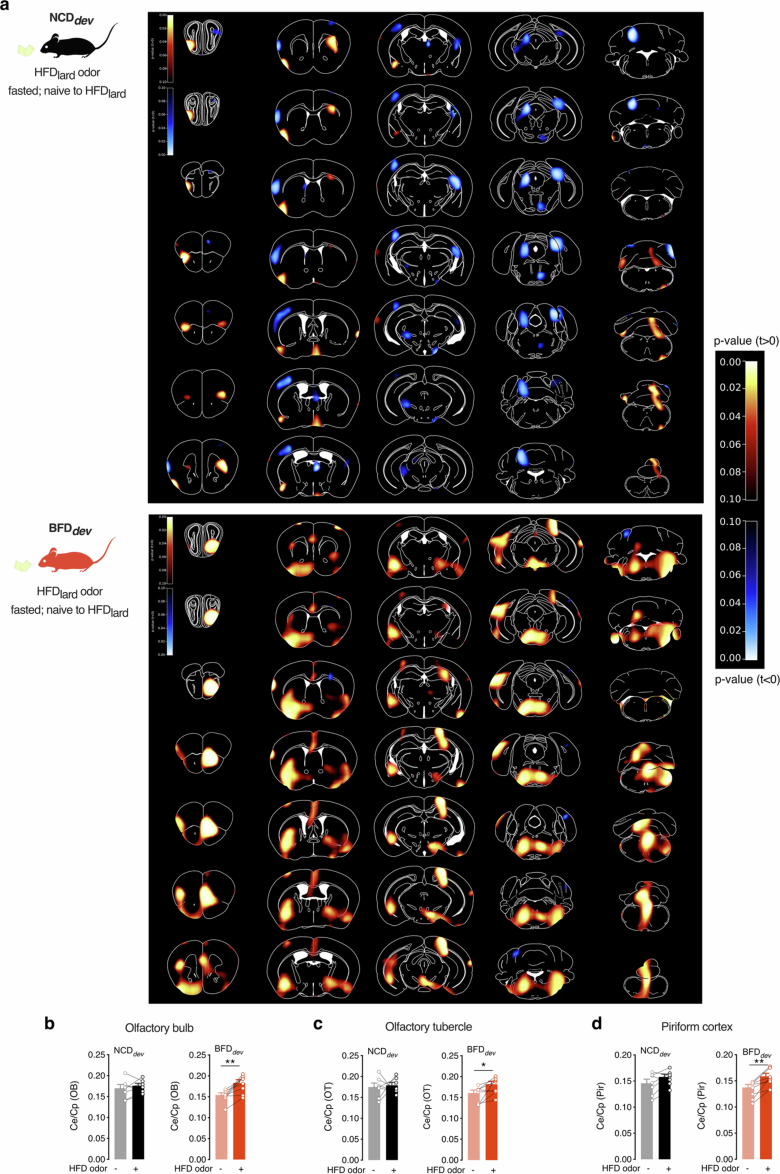
Developmental exposure to fat-related sensory cues alters central responses to HFD_lard_ odour. **(a)** Positron emission tomography measurement of glucose transport based on fluorodeoxyglucose (^18^FDG) kinetics in brains of NCD_*dev*_ or BFD_*dev*_ mice upon lard-based high-fat diet (HFD_lard_) odour exposure. The colour code on the representative PET images depicts the paired t-test value for each voxel. Relative increases in glucose metabolism upon HFD_lard_ odour exposure are colour-coded in yellow to orange, whereas decreases are colour-coded in blue. **(b-c)** HFD_lard_ odour-induced differential glucose transport based on ^18^FDG PET kinetics in: **(b)** the olfactory bulb (OB) [paired two-tailed t-test; n = 7,8; *P* = 0.0027], **(c)** the olfactory tubercle (OT) [paired two-tailed t-test; n = 7,8; *P* = 0.0253] and **(d)** the piriform cortex (Pir) [paired two-tailed t-test; n = 7,8; *P* = 0.0027]. Statistics are depicted as: paired t-tests. Bars represent mean value. Error bars represent SEM. * p ≤ 0.05, ** p ≤ 0.01. Source data

Developmental exposure to HFD leads to broad structural changes in the brain and alters hypothalamic homeostatic and mesolimbic reward-related circuits, thereby predisposing the offspring to maladaptive metabolic responses and hyperphagia^1,2,6,39–42^. To determine whether developmental exposure to fat-related sensory cues also alters central responses to food cues, we assessed alterations in brain activity in adult NCD_dev_ and BFD_dev_ mice. We measured glucose metabolism using fluorodeoxyglucose (^18^FDG) positron emission tomography imaging as a proxy for neuronal activity in response to HFD (Extended Data Fig. 7a). Upon acute HFD_lard_ odour exposure in mice naive to HFD_lard_, BFD_dev_ mice exhibited overall increased neuronal activity in several brain regions (Extended Data Fig. 7a). HFD_lard_ odour activated olfactory relevant regions such as the olfactory bulb, olfactory tubercle and piriform cortex in both groups, with greater responses observed in BFD_dev_ mice (Extended Data Fig. 7b–d). Additionally, HFD_lard_ odour exposure increased neuronal activity in dopaminergic reward-associated regions of the lateral nucleus accumbens shell (LAcbSh) (Fig. 3a) and the ventral tegmental area (VTA) (Fig. 3b) in BFD_dev_ mice, but not in NCD_dev_ mice.

**Extended Data Fig. 8:**
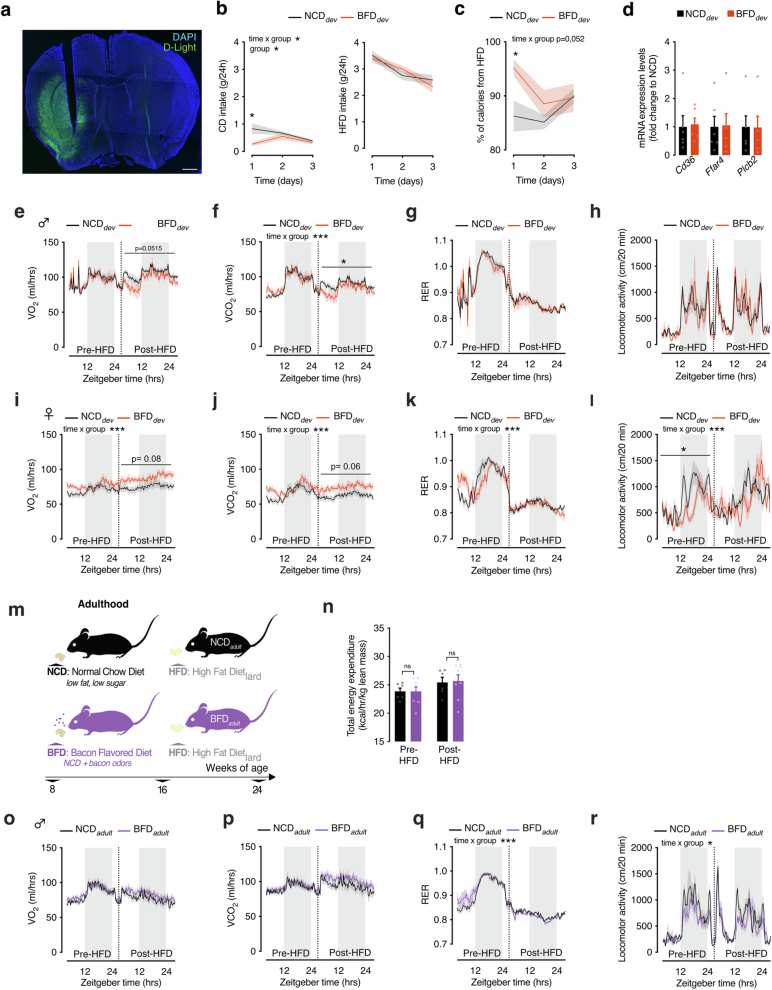
Exposure to fat-related sensory cues during adulthood does not influence diet-induced obesity. **(a)** Representative fibre placement and viral expression of dLight1.1, scale bar = 500 μm. **(b)** Intake of control diet (CD) or lard-based high-fat diet (HFD_lard_) during a two-choice diet preference test in adult mice developmentally exposed to normal chow diet (NCD_*dev*_) or bacon-flavoured diet (BFD_*dev*_) [two-way repeated measures ANOVA; Tukey’s post hoc; n = 13,10; *P* group = 0.0266; *P* interaction = 0.0354]. **(c)** Percentage of calories from HFD_lard_ during 3 days of two-choice diet preference test [two-way repeated measures ANOVA; Tukey’s post hoc; n = 13,10; *P* interaction = 0.0523]. **(d)** Relative expression of genes involved in fat-sensing in the tongue [Multiple unpaired two-tailed t-test; n = 6,6]. **(e-h)** Metabolic characterization upon the switch to HFD_lard_, *that is* pre- and post-HFD_lard_ feeding of NCD_*dev*_ and BFD_*dev*_ males: **(e)** VO_2_, **(f)** VCO_2_, **(g)** respiratory exchange ratio (RER), and **(h)** locomotor activity [two-way repeated measures ANOVA; n = 11,5; **(e)** Post-HFD *P* group= 0.0515; **(f)** *P* interaction = 0.0002, Post-HFD *P* group = 0.0488]. **(i-l)** Metabolic characterization upon the switch to HFD_lard_, *that is* pre- and post-HFD_lard_ feeding in NCD_*dev*_ and BFD_*dev*_ females: **(i)** VO_2_, **(j)** VCO_2_, **(k)** RER, and **(l)** locomotor activity [two-way repeated measures ANOVA; n = 14,8; **(i)** *P* interaction < 0.0001, **(j)** *P* interaction < 0.0001, Post-HFD *P* group = 0.0647; **(k)** *P* interaction < 0.0001, **(l)** *P* interaction < 0.0001, Pre-HFD *P* group= 0.0201]. **(m-r)** Metabolic characterization upon the switch to HFD_lard_, *that is* pre- and post-HFD_lard_ feeding in NCD_*adult*_ and BFD_*adult*_ males: **(n)** total energy expenditure [linear mixed-effects models; Holm–Bonferroni post hoc; n = 6,8], **(o)** VO_2_, **(p)** VCO_2_, **(q)** RER, and **(r)** locomotor activity [two-way repeated measures ANOVA; n = 7,8]. Statistics are depicted as two-way repeated measures ANOVA for repeated t-test and t-test horizontal bar represents group comparison. Bars represent mean value. Error bars represent SEM. * p < 0.05, *** p < 0.001. Source data

Because dopamine release in the LAcbSh encodes preference for and intake of dietary fat^43–45^, we wanted to investigate the effects of developmental exposure to fat odours on adult dopamine responses to food. We performed fibre photometry using the extracellular optical dopamine sensor dLight1.1 (ref. ^46^) in the LAcbSh (Fig. 3c and Extended Data Fig. 8a). We recorded dopamine release in the LAcbSh in fasted NCD_dev_ and BFD_dev_ adult mice in response to NCD, BFD or HFD_lard_ pellets. All dopamine fibre photometry experiments were performed in NCD_dev_ and BFD_dev_ mice on NCD acclimated to each diet, hence before the onset of any metabolic alterations. These experiments revealed that dopamine release in response to NCD was largely blunted in BFD_dev_ mice compared with NCD_dev_ (Fig. 3d). Furthermore, dopamine release upon BFD (Fig. 3e) or HFD_lard_ presentation (Fig. 3f) was similar between NCD_dev_ and BFD_dev_. Therefore, pre-HFD_lard_ (before the onset of metabolic alterations), BFD_dev_ mice show functional alterations in their central dopamine response to food characterized by a primed devaluation of dopaminergic response to NCD.

Given that dopamine responses to NCD are reduced in BFD_dev_ mice and that dopamine in the LAcbSh dictates preference and fat intake^43–45^, we next assessed whether the altered dopamine response translates into behavioural consequences. We performed a two-choice food preference test in adult NCD_dev_ and BFD_dev_ mice by exposing them to HFD_lard_ and a low-fat control diet (Fig. 3g). Compared with NCD_dev_, BFD_dev_ mice consumed a significantly higher percentage of calories from HFD_lard_ relative to a less palatable low-fat control diet during the first 24 h (Fig. 3g and Extended Data Fig. 8b,c). Of note, the enhanced HFD_lard_ preference observed in BFD_dev_ mice did not correlate with changes in the expression of the key fat taste receptors *Cd36* and *Gpr120* (*Ffar4)* nor *Plcb2*, an intracellular signalling pathway component critical for fat sensing (Extended Data Fig. 8d). Food intake was subsequently assessed under conditions in which mice were exclusively provided with HFD_lard_, replicating the experimental parameters utilized during metabolic phenotyping (Fig. 1n–u). Pre-HFD_lard_, both groups of mice consumed similar amounts of food (Fig. 3h). Similarly, total daily food intake of HFD_lard_ did not differ between NCD_dev_ and BFD_dev_ mice, either acutely (the first 2 weeks on HFD_lard_; Fig. 3i) or after long-term exposure (4–10 weeks on HFD_lard_; Fig. 3i). Collectively, these results reveal that developmental exposure to fat-related sensory cues influences first encounter feeding by shifting preferences towards HFD_lard_, while it does not alter total HFD_lard_ intake under exclusive HFD_lard_ feeding.

## Developmental exposure to fat-related odours impairs metabolic flexibility towards HFD

Our results suggest that the increased weight gain and adiposity in BFD_dev_ mice under HFD_lard_ is not triggered by increased food intake. Hence, we sought to further characterize the homeostatic responses to HFD feeding in adult BFD_dev_ mice using energy expenditure (EE) as a read-out of homeostatic control of energy metabolism. Before switching to HFD_lard_, EE was comparable between NCD_dev_ and BFD_dev_ groups, in both males (Fig. 3j) and females (Fig. 3k). In response to HFD_lard_ feeding, while EE increased in NCD_dev_ male mice (Fig. 3j), it decreased in BFD_dev_ male mice (Fig. 3j). EE also decreased in BFD_dev_ females compared with NCD_dev_ females after the switch to HFD_lard_ (Fig. 3k). Unlike EE, locomotion and respiratory exchange ratio were identical between the two groups after the switch to HFD_lard_ (Extended Data Fig. 8e–l). Notably, exposure to BFD exclusively during adulthood did not alter EE upon the switch to HFD_lard_ (Fig. 3l and Extended Data Fig. 8m–r), indicating that the decreased EE observed in BFD_dev_ is contingent on the sensory detection of fat-related cues during early life. As iBAT thermogenesis plays a central role in the regulation of EE in mice, we measured iBAT thermogenesis longitudinally in response to HFD_lard_ feeding (Fig. 3m). Pre-HFD_lard_, iBAT thermogenesis was similar between BFD_dev_ and NCD_dev_ mice. After switching to HFD_lard_, iBAT thermogenesis increased in both NCD_dev_ and BFD_dev_ mice (Fig. 3m). However, iBAT thermogenesis was lower in BFD_dev_ compared with NCD_dev_. This reduction in iBAT temperature was also accompanied by decreased expression of iBAT thermogenesis-related genes such as *Cidea* or *Pparg* (Fig. 3n). Therefore, we found that developmental exposure to HFD-related sensory signals alters metabolic flexibility and homeostatic responses to HFD in adult mice.

## Developmental exposure to fat-related sensory cues alters life-long AgRP neuronal activity dynamics

**Fig. 4:**
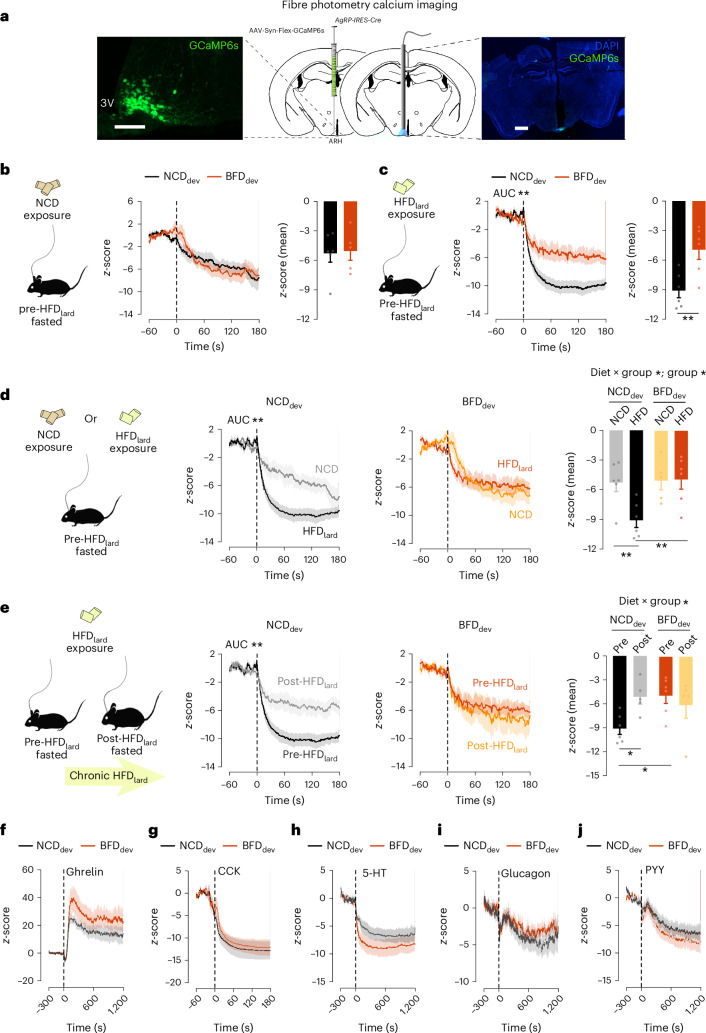
Developmental exposure to fat-related sensory cues primes an obese-like AgRP neuronal response before HFD_lard_-induced obesity. **a**, Schematic of viral injection of the calcium sensor GCaMP6s in the ARH and cannula implantation in AgRP-IRES-Cre mice for GCaMP6s-based fibre photometry. **b**,**c**, AgRP calcium dynamics and average *z*-score in response to NCD presentation pre-HFD_lard_ (unpaired two-tailed *t*-test; *n* = 6, 5) (**b**), non-naive HFD_lard_ presentation in NCD_dev_ and BFD_dev_ mice pre-HFD_lard_ (unpaired two-tailed *t*-test; *n* = 6, 6; *P* = 0.0071) (**c**). **d**, Comparison of the AgRP neuronal dynamics and average *z*-score in response to NCD and HFD_lard_ presentation in lean mice non-naive to HFD_lard_ in NCD_dev_ and BFD_dev_ (two-way ANOVA; uncorrected Fisher’s LSD post hoc; *n* = 6, 6; *n* = 5, 6, respectively; *P* group = 0.025; *P* interaction = 0.0417) (AUC_NCDdev_: unpaired two-tailed *t*-test; *n* = 6, 6; *P* = 0.0085). **e**, Comparison of the AgRP neuronal dynamics and average *z*-score in response to HFD_lard_ presentation following 4 weeks of HFD_lard_ feeding in NCD_dev_ and BFD_dev_ mice with average *z*-score (two-way ANOVA; uncorrected Fisher’s LSD post hoc test; *n* = 6, 5; *n* = 6, 5; *P* interaction = 0.0292) (AUC_NCDdev_: unpaired two-tailed *t*-test; *n* = 6, 5; *P* = 0.007). **f**–**j**, AgRP calcium dynamics pre-HFD_lard_ in response to intraperitoneal (i.p.) administration of ghrelin (*n* = 5, 7) (**f**), cholecystokinin (CCK) (*n* = 5,5) (**g**), serotonin (5-HT) (*n* = 5, 7) (**h**), glucagon (*n* = 4,7) (**i**) and peptide YY (PYY) (*n* = 5, 7) (**j**). Unpaired two-tailed *t*-test on the AUC (Extended Data Fig. 9h–l) (**f**–**j**). Statistics are depicted as for two-way repeated-measures ANOVA group comparison; unpaired *t*-test. Bars represent mean value. Error bars represent s.e.m. *P ≤ 0.05, **P ≤ 0.01. Source data

We next determined whether the exacerbated obesogenic responses and altered homeostatic responses induced by developmental exposure to fat-related sensory cues correlate with functional alterations in key homeostatic circuits. Based on their pivotal metabolic regulatory functions and their known implications in obesity-associated metabolic outcomes, we focused on Agouti-related peptide (AgRP) expressing neurons in the arcuate nucleus of the hypothalamus (ARH)^47–49^. AgRP neurons are highly active upon fasting and are pivotal homeostatic regulators of food intake, insulin sensitivity, EE and iBAT thermogenesis. To probe for the long-lasting influence of early life exposure to fat-related sensory cues on AgRP neurons, we monitored AgRP calcium dynamics in vivo in response to nutritionally relevant stimuli, including food as well as hunger and satiety hormones. To measure AgRP neuronal activity, fibre photometry was performed in adult NCD_dev_ and BFD_dev_ mice expressing the genetically encoded calcium indicator GCaMP6s in AgRP neurons (Fig. 4a).

**Extended Data Fig. 9:**
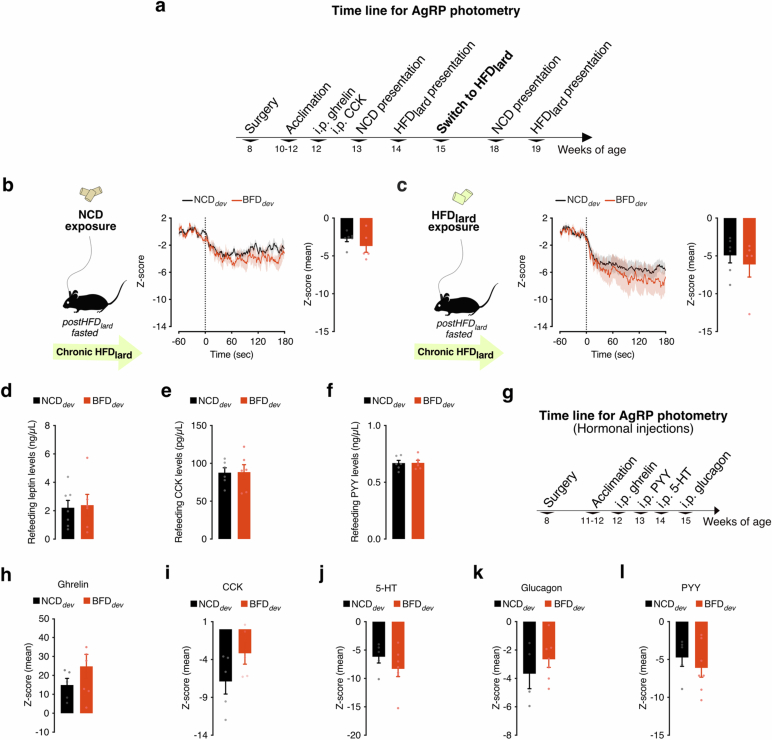
Developmental exposure to fat-related sensory cues primes an obese-like AgRP neuronal response. **(a)** Overview of the time line for recording AgRP neuronal dynamics using fibre photometry. **(b-c)** AgRP calcium dynamics and average Z-score following 4 weeks of HFD_lard_ in response to: **(b)** NCD presentation [unpaired two-tailed t-test; n = 6,5], **(c)** HFD_lard_ presentation [unpaired two-tailed t-test; n = 6,5]. **(d-f**) Circulating levels upon refeeding of **(d)** leptin [unpaired two-tailed t-test; n = 7,6], **(e)** cholecystokinin (CCK) [unpaired two-tailed t-test; n = 6,6] and **(f)** peptide YY (PYY) [unpaired two-tailed t-test; n = 6,6]. **(g)** Overview of the time line for recording AgRP neuronal dynamics upon intraperitoneal (i.p.) administration of hormones using fibre photometry. **(h-l)** Average Z-score of AgRP calcium dynamics in response to i.p. administration of **(h)** ghrelin [unpaired two-tailed t-test; n = 5,7], **(i)** cholecystokinin (CCK) [unpaired two-tailed t-test; n = 5,5], **(j)** serotonin (5-HT) [unpaired two-tailed t-test; n = 5,7], **(k)** glucagon [unpaired two-tailed t-test; n = 4,7] and **(l)** peptide YY (PYY) [unpaired two-tailed t-test; n = 5,7]. Statistics are depicted as: t-test. Bars represent mean value. Error bars represent SEM. Source data

Sensory detection of food-related cues rapidly and sharply decreases AgRP neuronal activity, and food ingestion sustains their inhibition^20,23–25,27^. In addition, studies have shown that obesity strongly impinges on AgRP neuronal activity dynamics^50,51^. In particular, the responsiveness of AgRP neurons to HFD is reduced in diet-induced obese mice^50,51^. To study the activity dynamics of AgRP neurons in our model, we first studied the AgRP calcium dynamics in fasted mice in response to food presentation. As AgRP neuronal inhibition in response to food is proportional to caloric content, we recorded AgRP calcium dynamics in adult NCD_dev_ and BFD_dev_ in response to both NCD and HFD_lard_ exposure. Of note, these photometry recordings have been performed in lean animals before chronic HFD_lard_ feeding but non-naive to HFD_lard_ to control for behavioural changes upon the first encounter with HFD_lard_ (Extended Data Fig. 9a). These experiments revealed that, as previously described, NCD presentation rapidly inhibited AgRP neurons (Fig. 4b). The AgRP neuronal response upon NCD presentation was identical in both groups (Fig. 4b). In striking contrast, AgRP neuronal inhibition upon HFD_lard_ presentation was largely blunted in BFD_dev_ compared with NCD_dev_ mice (Fig. 4c). In NCD_dev_ mice, the magnitude and dynamics of AgRP neuronal inhibition in response to food is proportional to the nutritional value, with HFD_lard_ presentation resulting in greater AgRP neuronal inhibition than NCD (Fig. 4d). In contrast, AgRP neuronal activity is identical in response to NCD and HFD_lard_ presentation in BFD_dev_ mice (Fig. 4d). Hence, developmental exposure to fat-related sensory cues impairs AgRP neuronal responses to dietary fat and outweighs the learnt responses to the food’s nutritional value. Collectively, these results revealed that adult mice developmentally exposed to fat-related sensory cues display functional dysregulations of AgRP neuronal responses that phenocopy those of adult HFD_lard_-fed obese mice while still lean and maintained on chow diet.

We then sought to investigate whether the ‘obese-like’ AgRP calcium dynamics in BFD_dev_ mice will be further affected by HFD_lard_-induced obesity. Hence, mice were switched to HFD_lard_ for 4 weeks and we repeated the fibre photometry recordings (post-HFD_lard_) (Extended Data Fig. 9a). These analyses revealed that, in line with previous reports, in NCD_dev_ animals, the inhibition of AgRP neuronal activity in response to HFD_lard_ is largely reduced in obese HFD_lard_-fed mice compared with lean mice (pre-HFD_lard_) (Fig. 4e and Extended Data Fig. 9b,c). In contrast, in BFD_dev_ mice, chronic HFD_lard_ feeding has no additional effects on AgRP neuronal inhibition in response to HFD_lard_ presentation (Fig. 4e and Extended Data Fig. 9b,c). Notably, the AgRP responses to HFD_lard_ in BFD_dev_ mice pre- and post-HFD_lard_ feeding are indistinguishable (Fig. 4e). Thus, early life exposure to fat-related sensory cues primes AgRP neuronal desensitization to dietary fat in ways that mirror that of diet-induced obese animals and that forego the onset of diet-induced metabolic alterations.

AgRP neuronal activity is modulated not only by food cues but also by hunger and satiety hormones^20,23,24,27,50,51^. Consequently, we next investigated whether the dampened AgRP neuronal response to HFD_lard_ could originate from changes in circulating levels of satiety hormones and/or altered AgRP neuronal responses to these hormones pre-HFD_lard_. Circulating levels of leptin, cholecystokinin (CCK) and peptide YY (PYY) after HFD_lard_ ingestion were similar between NCD_dev_ and BFD_dev_ mice (Extended Data Fig. 9d–f). We then assessed AgRP neuronal activity of NCD_dev_ and BFD_dev_ mice in response to the hunger-related hormone ghrelin and to key post-prandial AgRP neurons’ regulators (CCK, serotonin (5-HT), glucagon and PYY) (Extended Data Fig. 9g). AgRP neuronal activity did not differ between NCD_dev_ and BFD_dev_ mice in response to intraperitoneally (i.p.) administered ghrelin (Fig. 4f and Extended Data Fig. 9h), CCK (Fig. 4g and Extended Data Fig. 9i), 5-HT (Fig. 4h and Extended Data Fig. 9j), glucagon (Fig. 4i and Extended Data Fig. 9k) and PYY (Fig. 4j and Extended Data Fig. 9l). Therefore, developmental exposure to fat-related sensory cues led to a selective impairment in AgRP neuronal responses to dietary fat without affecting responses to key hormonal signals.

## Sensory programming of metabolism is dependent on sensory/caloric association

**Extended Data Fig. 10:**
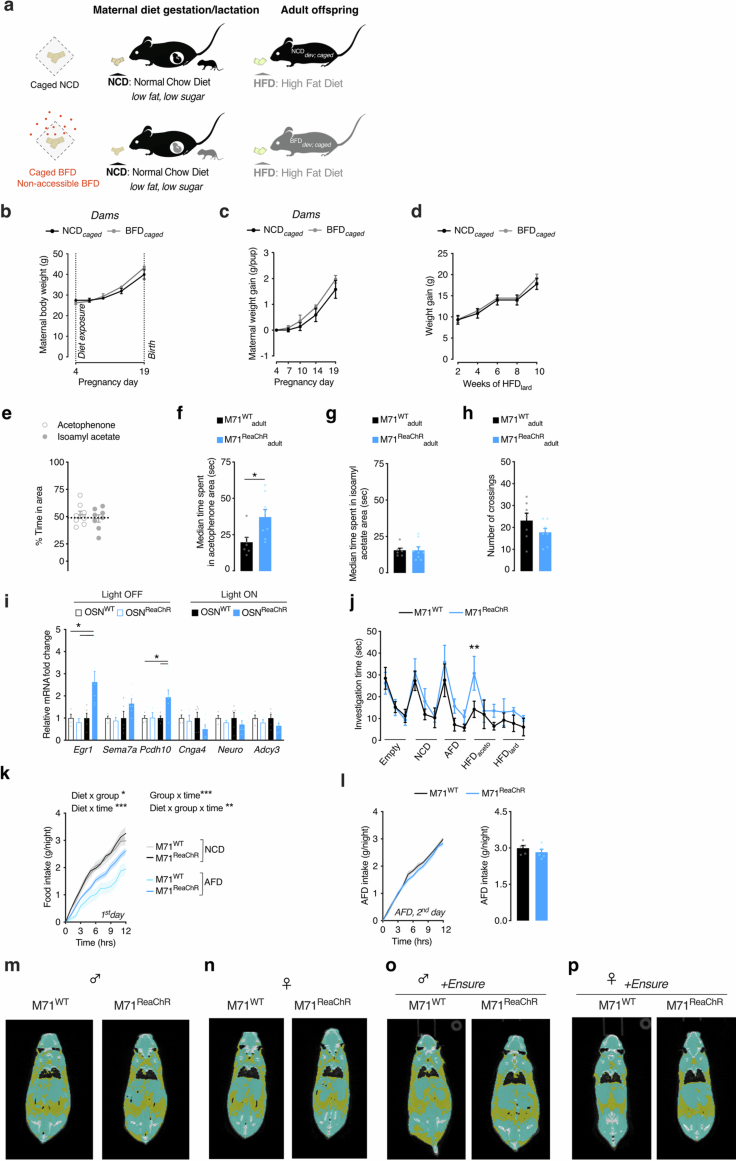
The sensory programming of metabolism is contingent on sensory/caloric association. **(a)** Paradigm of developmental exposure to odour cues in their home cage from inaccessible NCD (NCD_*dev;caged*_) or BFD (BFD_*dev;caged*_) feeding followed by lard-based high-fat diet feeding (HFD_lard_) during adulthood. **(b)** Maternal weight across gestation [two-way repeated measures ANOVA; n = 4,3]. **(c)** Maternal weight across gestation [two-way repeated measures ANOVA; n = 4,3]. **(d)** Body weight gain of male NCD_*dev;caged*_ or BFD_*dev;caged*_ offspring on HFD_lard_ [mixed-effects model; n = 16,15]. **(e)** Percentage of time spent in each area in C57BL/6NCrl mice [unpaired two-tailed t-test; n = 8,8]. **(f-h)** Two-chamber place preference test in adult mice expressing ReaChR in M71-expressing cells (M71^ReaChR^_adult_) and control littermates (M71^WT^_adult_) that underwent an optostimulation/food reward pairing protocol [unpaired two-tailed t-test; n = 7,8]. Median time spent **(f)** in acetophenone [*P* = 0.0186]. and **(g)** in isoamyl acetate area. **(h)** Number of crossings between the two areas of the experimental box. **(i)** Relative expression of olfactory sensory neuron (OSN) activity-dependent genes in P3 pups expressing ReaChR in OMP-expressing OSNs (OSN^ReaChR^) or wildtype OSNs (OSN^WT^) 60 min after optogenetic stimulation [two-way ANOVA; Tukey’s post hoc; n light off = 4,3; n light on= 5,6; *P Egr1* < 0.0001; *P Pcdh10* = 0.0196]. **(j)** Habituation-dishabituation test in adult mice with neonatal optogenetic activation of M71-expressing OSNs (M71^ReaChR^) and control littermates (M71^WT^). Mice are consecutively exposed to control (empty tea ball) and odours of normal chow diet (NCD), acetophenone-flavoured diet (AFD), high-fat diet flavoured with acetophenone (HFD_aceto_) and lard-based high-fat diet (HFD_lard_) placed in tea balls [two-way repeated measures ANOVA; Tukey’s post hoc; n = 5,4]. **(k)** Cumulative food intake of NCD and AFD upon first exposure during the dark cycle [three-way repeated measures ANOVA; n = 5,5; *P* diet:group = 0.0135; *P* diet:time < 0.001; *P* group:time < 0.001; *P* group:time:diet = 0.0014]. **(l)** Cumulative food intake of AFD upon first exposure during the second dark cycle of exposure [unpaired two-tailed t-test; n = 5,5]. **(m-p)** Representative CT images of **(m)** male and **(n)** female M71^ReaChR^ mice and control littermates M71^WT^ fed HFD_acetophenone_ during adulthood (related to Fig. 5o and q). **(o)** Male and **(p)** female M71^ReaChR+Ensure^ mice and control littermates M71^WT+Ensure^ fed HFD_acetophenone_ during adulthood (related to Fig. 5t and v). Statistics are depicted as two-way repeated measures ANOVA or mixed-effects model, group comparison. Bars represent mean value. Error bars represent SEM. * p ≤ 0.05, ** p ≤ 0.01, *** p ≤ 0.001. Source data

As in our model the bacon sensory cues are associated with the nutritive components of the ingested (flavoured) NCD, we sought to investigate if the sensory programming of obesity is contingent on the coupling with calorie ingestion. To unravel whether sensory stimulation alone is sufficient to prime long-term metabolic health, we repeated the developmental exposure to BFD in dams fed NCD but exposed to non-accessible BFD (Extended Data Fig. 10a). More precisely, we created a model of passive BFD exposure in NCD-fed dams by placing BFD in a metal mesh in the cage to expose the mice to its sensory signals uncoupled from its intake. Passive exposure to BFD (BFD_caged_) did not alter maternal weight gain or weight gain per pup (Extended Data Fig. 10b,c). Furthermore, passive exposure to BFD during development (BFD_dev;caged_) did not influence HFD_lard_-induced weight gain (Extended Data Fig. 10d). Altogether, these experiments suggest that the coupling of both sensory and nutritive food components is a prerequisite for the sensory programming of metabolism.

To substantiate the importance of sensory/caloric associations for the long-term susceptibility to obesity, we designed another model of developmental activation of sensory circuits independent of dietary manipulation. In mice, the neonatal period is critical for the experience-dependent regulation of sensory circuit development and responses later in life^16,17,52^. In particular, developmental acquisition of odour preference is dependent upon the activity of OSNs which reside in the main olfactory epithelium^16,17,52^. Accordingly, we developed a non-invasive optogenetic-based model of neonatal activation of a defined olfactory circuit to assess the subsequent consequences on odour-evoked behaviours, food preference and obesity development. Having identified acetophenone as a volatile highly enriched in BFD and HFDs (Fig. 2m), we selectively activated OSNs expressing olfactory receptor 151 (Olfr151, also known as M71), a primary olfactory receptor for acetophenone^53^. We targeted the red-shifted variant of channelrhodopsin (ReaChR), which allows non-invasive light stimulation^54^ of M71-expressing OSNs in pups. This experimental design enables to investigate the role of neonatal activation of an acetophenone-responsive circuit on long-term behavioural and obesogenic outcomes, independent of maternal diet or direct acetophenone exposure. By selectively activating this sensory circuit during early postnatal development, we aimed to establish a proof-of-concept experiment to isolate the effects of early sensory circuit activation on lasting behavioural and metabolic trajectories.

**Fig. 5:**
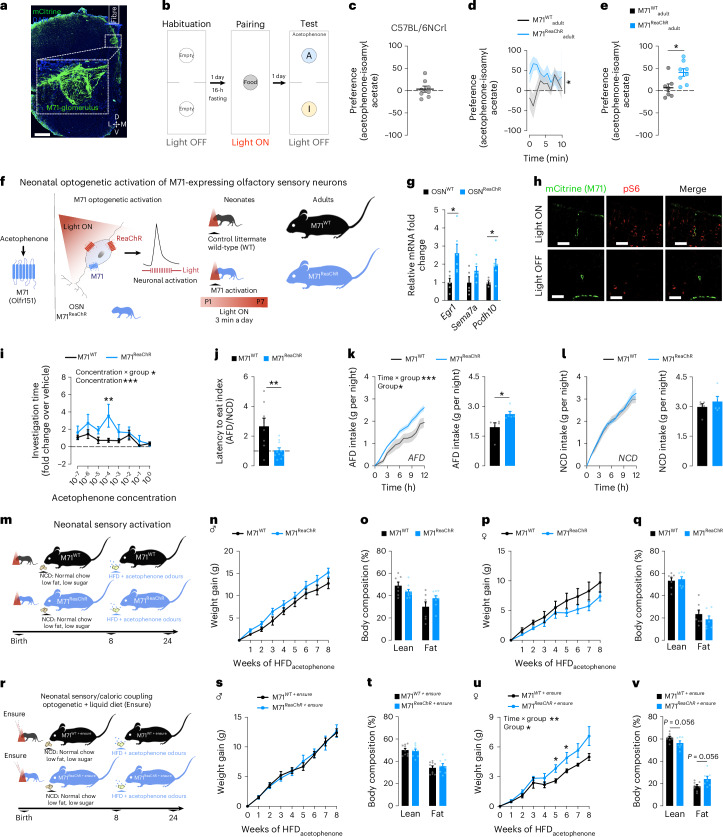
The sensory programming of metabolism is contingent on sensory/caloric association. **a**–**e**, Optogenetic activation of M71 glomeruli in M71-IRES-Cre:R26-LSL-ReaChR-mCitrine adult mice. Representative image of fibre placement above a M71 glomerulus in the olfactory bulb; scale bar, 300 μm (green, mCitrine (M71); blue, 4,6-diamidino-2-phenylindole (DAPI)) (**a**). Schematic of the experimental design to pair M71 photostimulation with a food reward in adult mice (**b**). The implantation of artificial memory was assessed using a two-chamber place preference test, with each chamber containing either acetophenone or a control odour (isoamyl acetate). Place preference test in C57BL/6NCrl mice (*n* = 8) (**c**). Odour preference in mice expressing ReaChR in M71 cells (M71^ReaChR^_adult_) and control littermates (M71^WT^_adult_). Odour preference test (two-way repeated-measures ANOVA; Sidak’s post hoc; *n* = 7, 8; *P* group = 0.0151) (**d**) and average preference of M71^ReaChR^_adult_ and M71^WT^_adult_ mice (unpaired two-tailed *t*-test; *n* = 7, 8; *P* group = 0.015) (**e**). **f**, Paradigm of neonatal optogenetic activation of M71-expressing OSNs. **g**, Gene expression of OSN activity-dependent genes in P3 pups expressing ReaChR in OMP-expressing OSNs (OSN^ReaChR^) or WT OSNs (OSN^WT^) 60 min after optogenetic stimulation (multiple unpaired two-tailed *t*-test; *n* = 5, 6; *P Egr1* = 0.0184; *P Pcdh10* = 0.0337). **h**, Representative images of S6-phosphorylation (pS6) in the main olfactory epithelium in P5 pups expressing ReaChR in M71 OSNs (M71^ReaChR^) and control littermates (M71^WT^) (green, mCitrine (M71); red, pS6), scale bar, 20 μm. **i–v**, Behavioural and metabolic analysis of adult mice optogenetically stimulated during neonatal life. Investigation time of progressively increasing concentrations of acetophenone normalized to vehicle (two-way repeated-measures ANOVA; Sidak’s post hoc; *n* = 6, 5; *P* concentration < 0.0001; *P* interaction = 0.0349) (**i**). Latency to eat AFD normalized to NCD following overnight fast (unpaired two-tailed *t*-test; *n* = 8, 11; *P* = 0.0048) (**j**). Cumulative food intake of AFD upon first exposure during the dark cycle (time curve: two-way repeated-measures ANOVA; Sidak’s post hoc; *n* = 5, 5; *P* group = 0.0364; *P* interaction = 0.0003) (total AFD intake, unpaired two-tailed *t*-test; *n* = 5, 5; *P* group = 0.0339) (**k**). Cumulative NCD intake (time curve, two-way repeated-measures ANOVA; *n* = 5, 5) (total NCD intake, unpaired two-tailed *t*-test; *n* = 5, 5) (**l**). Paradigm of neonatal optogenetic activation of M71-expressing OSNs and subsequent feeding of HFD_acetophenone_ (**m**). Metabolic phenotyping of adult offspring optogenetically stimulated during the first week of life and fed a HFD_acetophenone_ during adulthood. Body weight gain of male offspring on HFD_acetophenone_ (two-way repeated-measures ANOVA; *n* = 14, 12) (**n**). Body composition of males following HFD_acetophenone_ (two-tailed Wilcoxon rank-sum test; *n* = 7, 9) (**o**). Body weight gain of female offspring on HFD_acetophenone_ (two-way repeated-measures ANOVA; *n* = 8, 10) (**p**). Body composition of females following HFD_acetophenone_ (two-tailed Wilcoxon rank-sum test; *n* = 7, 7) (**q**). Schematic of the neonatal sensory/caloric coupling paradigm: light stimulation was coupled to the intake of high-calorie liquid diet Ensure in pups expressing ReaChR in M71-expressing OSNs (M71^ReaChR+Ensure^) and control littermates (M71^WT+Ensure^) (**r**). Metabolic phenotyping on HFD_acetophenone_. Body weight gain of male offspring on HFD_acetophenone_ (mixed-effects model; *n* = 10, 11) (**s**). Body composition of males following HFD_acetophenone_ (two-tailed Wilcoxon rank-sum test; *n* = 9, 8) (**t**). Body weight gain of female offspring on HFD_acetophenone_ (mixed-effects model; Tukey’s post hoc; *n* = 9, 9; *P* group = 0.0182; *P* interaction = 0.0036) (**u**). Body composition of females following HFD_acetophenone_ (two-tailed Wilcoxon rank-sum test; *n* = 8, 7; *P* = 0.056) (**v**). Statistics are depicted as: for two-way ANOVA, group comparison and time x group interaction; for multiple unpaired *t*-test and Wilcoxon rank-sum test, horizontal bars represent group comparison. Bars represent mean value. Error bars represent s.e.m. *P ≤ 0.05, **P ≤ 0.01, ***P ≤ 0.001. Source data

To first validate that optogenetic activation of M71 can resemble exposure to acetophenone in the absence of sensory experience, we conducted experiments in adult mice expressing ReaChR in M71-expressing neurons (M71^ReaChR^_adult_) (Fig. 5a–e and Extended Data Fig. 10e–h). To determine if optogenetic stimulation of M71 can guide future olfactory behaviour in response to acetophenone, we paired photostimulation of M71 glomeruli in the olfactory bulb (where OSN-expressing M71 axons converge) with a food reward in the form of a sucrose-coated HFD_lard_ pellet (Fig. 5a,b). We then subjected the mice to a two-chamber odour preference test, exposing them to acetophenone and isoamyl acetate as control odour (Fig. 5b). In the absence of experimental intervention, mice typically showed no preference between the two odours (Fig. 5c and Extended Data Fig. 10e). In contrast, pairing M71 stimulation with a food reward triggered a preference for the acetophenone side in M71^ReaChR^_adult_, while M71^WT^_adult_ control mice showed no preference between the two odours (Fig. 5d,e and Extended Data Fig. 10f–h). These experiments confirm that M71 photostimulation effectively mimics activation of acetophenone-sensitive circuits in the absence of real odour experience.

To activate M71-expressing OSNs during neonatal life, the snouts of pups expressing ReaChR in M71 neurons (M71^ReaChR^) and their control Cre-negative littermates (M71^WT^) were light-stimulated from postnatal day 1 to 7 (Fig. 5f), which covers the olfactory critical period pivotal for developmental acquisition of odour preference^16,17,52^. To validate this approach in pups, a red light was placed over the snout of pups expressing ReaChR in all OSNs (OSN^ReaChR^) at postnatal day 3 before assessing the resulting regulation of activity-dependent genes (Fig. 5g and Extended Data Fig. 10i). Expression of *Egr1* and *Pcdh10* increased upon light stimulation in OSN^ReaChR^ pups, but not in light-stimulated Cre-negative littermates (OSN^WT^) or in OSN^ReaChR^ non-light-stimulated pups (Fig. 5g and Extended Data Fig. 10i), demonstrating specific and selective light-induced activation of OSNs. Furthermore, light stimulation activated M71-expressing neurons in the MOE of ReaChR-expressing pups (M71^ReaChR^), as indicated by S6 phosphorylation, an effect not seen in Light OFF control animals (Fig. 5h). Thus, this model allows for acute, selective and non-invasive activation of olfactory circuits in neonates.

We first investigated the influence of this neonatal optogenetic activation of M71-expressing OSNs on the long-term acetophenone preference. Adult mice that underwent M71 optogenetic stimulation during early life (M71^ReaChR^) exhibited a preference for acetophenone as revealed by an increased investigation time of defined acetophenone concentrations (Fig. 5i). Furthermore, in an olfactory habituation-dishabituation test, M71^ReaChR^ mice exhibited increased investigation time for a HFD flavoured with acetophenone (HFD_acetophenone_) (Extended Data Fig. 10j). To further assess the influence of M71 neonatal activation on feeding-related behaviour in adulthood, we exposed mice to NCD flavoured with acetophenone odour (AFD). Latency to eat AFD decreased in adult M71^ReaChR^ compared with M71^WT^ animals (Fig. 5j), demonstrating that neonatal optogenetic activation of M71-expressing OSNs is sufficient to programme odour preference in adulthood. Remarkably, M71^ReaChR^ mice ate significantly more AFD (Fig. 5k), whereas intake of NCD was identical in both groups (Fig. 5l and Extended Data Fig. 10k). Further analysis of feeding behaviour revealed that increased AFD intake in M71^ReaChR^ mice occurred only acutely (the first day) and that both groups ingested a similar quantity of AFD on the second day (Extended Data Fig. 10l). Thus, selective neonatal stimulation of olfactory circuits primes acute food preference similar to the HFD preference observed in BFD_dev_ mice_._ Together, these findings reveal that activation of sensory circuits during development is sufficient to gate feeding preference in adulthood.

To evaluate whether neonatal optogenetic activation of a distinct sensory circuit can influence the onset of obesity in adulthood, adult M71^ReaChR^ and Olfr51^WT^ mice were fed HFD_acetophenone_ (Fig. 5m). Neonatal M71 optogenetic activation did not significantly influence diet-induced weight gain (Fig. 5n) or adiposity neither in males (Fig. 5o and Extended Data Fig. 10m) nor in females (Fig. 5p,q and Extended Data Fig. 10n). Thus, activation of sensory circuits alone programmes food and odour preference, but is not sufficient to exacerbate the obesogenic responses to HFD_acetophenone_.

In contrast to our BFD-based model of developmental exposure to fat-related odours, optogenetic-based activation of sensory circuits is uncoupled from caloric intake. To investigate whether the sensory programming of metabolism relies on the association between sensory input and caloric intake, we paired neonatal M71 optogenetic activation with caloric ingestion. This sensory/caloric coupling was achieved by allowing the pups to lick the calorie-dense liquid diet Ensure during optogenetic stimulation (M71^ReaChR+Ensure^ versus M71^WT+Ensure^) (Fig. 5r). Crucially, as pups consume only a small fraction of their daily caloric intake during the optogenetic procedure, this model enables sensory-caloric coupling without triggering neonatal overeating.

Coupling neonatal M71 optogenetic stimulation to caloric intake did not affect HFD_acetophenone_-induced weight gain (Fig. 5s) or adiposity (Fig. 5t and Extended Data Fig. 10o) in males. In contrast, M71^ReaChR+Ensure^ females showed exacerbated weight gain (Fig. 5u; interaction sex × developmental diet × time, *P* = 0.02) and adiposity (Fig. 5v and Extended Data Fig. 10p; interaction sex x developmental diet: *P* = 0.03) compared with M71^WT+Ensure^ control mice. Collectively, these results highlight that the sensory programming of metabolism is contingent on sensory/caloric association and further corroborate sexual dimorphism in these responses.

## Discussion

Extensive research over the past three decades has established that maternal dietary habits can profoundly shape the health trajectories of their offspring. Yet, the primary focus has remained on the caloric and nutritional composition of the diet, while the role of food chemosensory cues has been neglected. Here, we demonstrate that exposure to the non-nutritive sensory components of HFD during development is sufficient to induce long-lasting metabolic dysregulation and prime obesity similar to the deleterious effects triggered by obese or HFD-fed dams^1–5^.We reveal that early life exposure to fat-related sensory cues is sufficient to shift the responses of reward-related mesolimbic dopaminergic and homeostatic AgRP circuits towards response characteristics of obese animals. Altogether, we identify a novel pivotal mechanism through which maternal diet primes long-term metabolism and programmes obesity in the offspring.

Our study provides evidence that maternal obesity per se and its associated adverse metabolic outcomes are not solely responsible for the metabolic programming triggered by maternal HFD feeding. Our results are in line with previous work revealing that offspring of lean HFD-fed dams have an increased risk for metabolic dysregulation^1,3,4^. Notably, studies in non-human primates revealed that HFD consumption in diet-resistant lean mothers triggered metabolic alterations in the offspring^7,8^. Conversely, switching obese non-human primate mothers to a chow diet exclusively during pregnancy reversed peripheral alterations in their offspring^7^, further corroborating that diet itself plays a key programming role independent of maternal health status.

Maternal HFD consumption affects dopamine circuits and induces a preference for HFD in the offspring^41,55,56^. While the diet-induced remodelling of dopamine responses has been largely attributed to the hyperlipidaemic and hypercaloric aspects of palatable foods, our study alternatively reveals another pathway by demonstrating that non-nutritive sensory cues influence the mesolimbic reward pathway in adulthood. Critically, the blunted dopamine release observed in the LAcbSh and the shift in preference for HFD over a less palatable chow diet have recently been reported in adult HFD-fed mice^50^. The dampened dopamine response observed in BFD_dev_ mice occurred in lean NCD-fed animals, suggesting that these alterations occur independently of metabolic changes. Consistent with our results, other studies have reported that altered dopamine responses to food and subsequent changes in feeding behaviour can occur in the absence of weight gain or metabolic alterations in adult animals fed HFD^50,57,58^. We show that the presence of fat-related sensory cues in the developmental diet not only durably altered dopamine responses to food, but also gated food discrimination and preference. Our neonatal M71 optogenetic model further emphasizes the importance of sensory circuit activation during development in shaping odour and food preference in adulthood. Although we attribute the results of the food preference test to a preference, they may also reflect reduced or absent neophobia (the reluctance to consume unfamiliar foods), which affects food choice, particularly in children^59^. Aligned with other studies^60^, the preference for HFD_lard_ occurs over the course of the first day of exposure in control animals, while mice exposed to BFD during development show an almost immediate preference for HFD_lard_ over the control diet. Further supporting the involvement of neophobia, the effects on feeding preference were acute (≈24 h), indicating they arise from behavioural differences during initial encounters. Of note, while pups begin ingesting solid food in the second postnatal week, raising the possibility of pre-weaning BFD effects, our diet-independent neonatal optogenetic model rules out any major contribution of pup-driven BFD intake to the observed phenotypes.

In contrast to observations in adult obese mice^50,51^, our model of developmental exposure to fat-related sensory cues shows that the diminished dopamine release in response to NCD was not accompanied by changes in AgRP neuronal activity. In fact, NCD exposure induced a rapid inhibition of AgRP neuronal activity in both groups, in line with the previously described sensory inhibition of these neurons^20,23–25,27^. This suggests that food sensory experiences in early life independently influence mesolimbic dopamine and AgRP hunger circuits to gate preference towards HFD and trigger metabolic alterations, respectively. Despite similar responses to NCD exposure, BFD_dev_ mice exhibited AgRP neuronal desensitization to dietary fat, a phenomenon typically observed in mice chronically fed HFD^50,51^. Our findings align with previous studies demonstrating that genetic disruption of AgRP neuronal function leads to increased EE^61–65^. Similarly, neonatal ablation of AgRP neurons^66^ as well as deletion of either the entire AgRP neuronal population or specific subpopulations^67,68^ elevate EE, further emphasizing the critical role of AgRP neurocircuits in the regulation of energy metabolism and body weight control. Remarkably, early life sensory cues can outweigh the profound effects on AgRP neurons of diet-induced obesity during adulthood, which had no additional detrimental consequences on these neurons. While maternal HFD feeding has a well-known negative influence on the development of AgRP neurocircuits^1,39,42^, the blunted responses to HFD did not seem to derive from general dysfunction of AgRP neurons, as they responded appropriately to NCD exposure and peripheral hormones. Consistent with the altered responses to HFD centrally and metabolically, reduced food cue-induced control of feeding behaviour is associated with overweight and obesity susceptibility in rodents and humans^69–73^. Considering the numerous central and peripheral pathways involved in the regulation of energy homeostasis, further studies will be required to define whether early life exposure to fat sensory cues influences processes beyond those described in this study. Collectively, our findings demonstrate that fat sensory cues during development are sufficient to mimic the deleterious effects of HFD-induced obesity on AgRP neurons and resulting metabolic alterations.

Recent investigations of the regulation of AgRP neuronal activity dynamics revealed the critical importance of learning processes^20,23–25,60,74,75^. Consumption of a novel food in a single trial is sufficient to condition AgRP responses proportionally to the caloric content upon exposure to sensory food cues^20^. In adult mice, this learnt sensory regulation of AgRP neurons is dependent on food consumption and the subsequent activation of chemosensory pathways in the intestine^76,77^. Here, we provide evidence for the importance of learnt sensory/caloric coupling mechanisms during the developmental period, likely mirroring what is occurring during adulthood. Our study indicates that developmental exposure to fat sensory cues coupled with a NCD might pre-empt the learnt AgRP neuronal response to HFD_lard_ during adulthood, resulting in maladaptive central and peripheral responses to an obesogenic diet. Furthermore, we demonstrate that the sensory programming of metabolism necessitates calorie ingestion. While real (caged food) and artificially implanted (neonatal optogenetics) food sensory memories during development did not influence diet-induced obesity when uncoupled from calorie intake, reintroducing calorie ingestion during neonatal M71 activation led to detrimental metabolic consequences. Remarkably, a short, daily artificial activation of M71 paired with caloric intake during the first days of life was sufficient to substantially exacerbate obesity and adiposity. In contrast, neonatal M71 activation, when uncoupled from caloric intake, did not induce metabolic alterations but was sufficient to influence odour and feeding preferences in adulthood. These findings suggest a model wherein food sensory cues during development gate feeding preferences independently of caloric intake, whereas the sensory programming of metabolism requires the coupling of sensory cues with calories. Given that both HFD and BFD emit a complex array of volatiles beyond acetophenone, our models of acetophenone exposure trough maternal diet and M71 neonatal activation serve as a proof of concept to validate the role of HFD-derived odorants in sensory modulation of metabolism, rather than delineating the primary molecular pathway mediating this effect. Future studies are required to dissect the intricate odour profile of fatty foods and elucidate the specific volatiles and olfactory receptors contributing to long-term obesogenic outcomes.

In mice, the acquisition of innate odour recognition is particularly influenced by sensory experience during early postnatal development^16,17,52^. Notably, OSN activity during this period regulates the expression of axon-sorting molecules, which in turn directly influence their development and life-long function^16,17,52^. Consistent with this, our optogenetic stimulation of M71 led to increased expression of axon-sorting molecules, including *Pcdh10*. Previous studies have demonstrated that OSN activity during this critical period, and the resulting modifications in the wiring of the glomerular map, have long-lasting effects on odour-evoked behaviours for odours encountered during development^16–19^. Accordingly, optogenetic stimulation of M71 olfactory circuits during the first week of life was sufficient to gate odour and feeding preference. Furthermore, neonatal M71 activation coupled with caloric intake and exposure to BFD during lactation triggered metabolic alterations only in females, highlighting that postnatal development may be a critical period for sensory programming of metabolism. This finding suggests that the sex-specific windows of sensitivity in models of maternal under- or over-nutrition also extend to the sensory programming of metabolism^78^. Although we did not observe sexual dimorphism in the sensory-induced programming of metabolism by bacon sensory cues when exposed throughout the developmental period, female mice exhibited heightened sensitivity to other paradigms of developmental food sensory manipulations. These sex-specific effects might arise from numerous factors, including sexual dimorphism in olfactory signalling^79^ and the sex-specific expression of distinct olfactory receptors in the main olfactory epithelium^80,81^. Further studies are required to clarify how sex shapes metabolic sensory programming, including effects on OSN activity and axon-sorting molecules. As experience drives sex differences in pheromone-sensing neurons of the vomeronasal organ^82^, the main olfactory epithelium may exhibit similar sex-dependent responses to specific food-related odorants. In addition, given that each diet has a complex odour profile composed of a large variety of volatiles, the lack of metabolic alterations in males exposed to acetophenone suggests that other volatiles may play a more prominent role in mediating the effects in males. Critically, the finding that developmental exposure to a single HFD_lard_-derived volatile sets the stage for obesity in adulthood further highlights the crucial importance of early food sensory experiences.

This study expands on previous work in human and animal models demonstrating that early chemosensory transmission through maternal diet can dictate feeding habits until adulthood towards a preference for the odours to which the embryos and neonates were exposed during development^12,15–19^. As the olfactory and gustatory systems are among the first senses to develop, food sensory experiences already begin in the amniotic environment in both humans and rodents. This early chemosensory transmission likely confers a notable evolutionary advantage by serving as teaching signals to guide individuals towards safe food choices while helping them avoid potentially toxic options. However, our study indicates that perinatal odour and flavour learning not only transmits food preferences to subsequent generations but can also have detrimental consequences for their metabolic health. In light of the large number of women consuming a HFD during pregnancy and lactation, these insights are important as they suggest that even a healthy weight mother, when exposed to an unhealthy fat-rich diet and despite apparent optimal health status, might put her offspring at higher obesity risk later in life. These findings might have far-reaching implications related to the long-term consequences of the consumption of flavouring agents during pregnancy and lactation. Given the rising prevalence of food additives in modern processed diets, our study raises the possibility that exposure to artificial flavours during development might create a mismatched association between food sensory signals and expected calorie content that might programme long-lasting inappropriate physiological and behavioural responses later in life. In this context, acetophenone, which we found to be sufficient to induce obesity in females when exposure occurs during development, is naturally present in foods, but is also an FDA-approved food flavouring agent widely used in industry. These findings, therefore, raise important questions about their translational relevance for human health and behaviour. In summary, our study reveals that the early life sensory environment exerts a long-lasting influence on the regulation of central responses to food cues and metabolic health and that an exacerbated response to an obesogenic diet can be primed by the mere exposure to fat-related sensory cues during development.

## Methods

### Animal care

Animal care, handling, and experimental procedures were conducted in compliance with protocols approved by local government authorities (Bezirksregierung Köln). Permission to maintain and breed mice was issued by the Department for Environment and Consumer Protection, Veterinary Section, Cologne, North Rhine-Westphalia, Germany. All mice were housed in individually ventilated cages at 22–24 °C at constant humidity (50–70%) with a 12-h light–dark cycle with ad libitum food and water unless otherwise stated for experimental conditions.

### Mouse models

C57BL/6NCrl mice were purchased from Charles River. AgRP-IRES-Cre mice (JAX stock, #012899)^65^, M71-IRES-Cre (JAX stock, #006677)^83^, OMP-Cre (JAX stock, #006668)^83^ and R26-LSL-ReaChR-mCitrine (JAX stock, #026294)^54^ were originally obtained from The Jackson Laboratory. Cre lines were maintained heterozygous through breeding to wild-type C57BL/6NCrl (Charles River), while R26-LSL-ReaChR-mCitrine mice were maintained as homozygous stock breeding at the Max Planck Institute for Metabolism Research.

### Diets

All diets used were obtained from ssniff Spezialdiäten (ssniff). NCD (ssniff R/M-H-Phytoestrogenarm) was used as the control maintenance diet and contained 57% calories from carbohydrates, 34% from protein and 9% from fat. The BFD, the PB-FD and AFD are isonutritional diets to NCD, containing 0.15% artificial flavours (Elli’s Aromen; bacon, AS10301; peanut butter, AS10312) or 1% acetophenone (AFD, used in behavioural tests). HFDs used in this study are: HFD_lard_ (ssniff EF acc. D12492 (I) mod, which contains 21% calories from carbohydrates, 19% from protein and 60% from fat derived from pork lard); HFD_butter_ (ssniff EF acc. D12492 (I) mod, which contains 21% calories from carbohydrates, 19% from protein and 60% from fat derived from butter) and HFD_acetophenone_ containing 0.25% acetophenone in HFD (HFD_lard_; ssniff EF acc. D12492 (I) mod). The peanut butter-based HFD (HFD_peanut-butter_) containing 34.9% kcal from fat was produced in-house by mixing 40% organic peanut butter (Bio Erdnussmus, dm-Drogerie Markt) with 60% NCD (ssniff R/M-H-Phytoestrogenarm) previously ground using a Koenic food blender. Feeding preference tests were performed using control diet (CD; ssniff EF acc. E15748) containing 67% calories from carbohydrates, 20% of protein, and 13% of fat.

### Paradigm of developmental exposure to fat-related sensory cues through the diet

C57BL/6NCrl mice were ordered from Charles River between 5 and 6 weeks old and allowed to acclimate in a group setting of 4–5 mice per cage for 2–3 weeks. To minimize the increased risks of pup mortality reported in primiparous C57BL/6N mice, experimental cohorts of animals were generated using pregnancy-experienced females that have already undergone one pregnancy at 8 weeks old. Mice were paired one male to one female for breeding (1:1 breeding) to generate experimental cohorts. Male breeders were C57BL/6NCrl or AgRP-IRES-Cre heterozygotes for photometry experiments. Males were removed upon first signs of visible pregnancy and single caged. Females taking longer than 19 days to show visible signs of pregnancy were excluded. At postnatal day 1 (P1; day of birth considered as P0) pups were removed from the cage and tissue collected. One week later, males were paired back with the same females in a 1:1 breeding scheme. The presence of a vaginal plug was checked each morning and used to time pregnancy. The presence of a vaginal plug was designated as gestational day (GD) 0.5 and female mice lacking vaginal plugs for 7 days were excluded. At GD 4.5, the males were removed from the breeding cage and the diet of the dam was switched as appropriate to either BFD, PB-FD, AFD or remained on NCD. Dams were maintained on their respective diets until weaning of the pups at postnatal day (P) 21. Litter size was adjusted to ensure adequate and standardized nutrition until weaning. Litters containing more than eight pups were culled down to eight pups at P1 and only these litters or those with six to eight pups naturally were used for future experiments. Offspring from a minimum of two separate litters per group were used for every experiment.

### Paradigm of developmental exposure to BFD during gestation or lactation selectively

The exact same experimental paradigm as described above was followed with the differences that dams were either exposed exclusively to BFD from GD 4.5 until birth (BFD_ges_; exposure during gestation only) or from birth (P0) until weaning (P21) (BFD_lac_; exposure during lactation only).

### Paradigm of developmental exposure to fat-related sensory cues (‘caged BFD’)

For caged food cohorts, the accessible diet remained NCD for the entire duration but a wire mesh containing 100 g of either NCD or BFD was placed into the bottom of the food rack and replaced every other day. Breeding strategy and litter size controls were performed as detailed above.

### Metabolic phenotyping: time line

#### Maternal metabolic phenotyping

Dams were weighed every 3 days during gestation and lactation. At weaning of the pups, body composition was analysed using micro-CT and mice were fasted overnight and blood was collected for HOMA-IR measurements. For LC–MS-based analysis of the hydrophobic (lipids fractions), 12 days after giving birth, milk was collected as described below as well as trunk blood upon killing.

#### Offspring metabolic phenotyping

Pups were weaned at P21 onto their respective diets and were weighed weekly throughout their lifespan. An insulin tolerance test (ITT) and body composition pre-HFD were performed at 6 and 8 weeks of age, respectively. At 8 weeks of age, animals from both groups were switched onto a HFD (post-HFD). At 12 weeks old (4 weeks on HFD), an ITT post-HFD was performed. At 16 weeks of age, resting blood glucose measurements were taken using a handheld glucometer from venous tail blood. At 18 weeks old, another micro-CT scan was performed for body composition analysis and body length measurements were taken from snout to anus using a ruler.

#### Offspring metabolic phenotyping (M71^ReaChR^)

Metabolic phenotyping of mice optogenetically stimulated during neonatal life was performed as described above but mice were fed an HFD enriched with acetophenone (0.25%).

#### Adult cohort metabolic phenotyping

Mice for adult cohorts were purchased from Charles River at 5–6 weeks old, housed four mice per cage and allowed to acclimate for 2–3 weeks in the animal facility. At 8 weeks old, mice were changed to BFD or maintained on NCD. Mice were then metabolically phenotyped according to a similar schedule to offspring mice. In short, at 16 weeks old, mice of both groups were switched onto HFD (HFD_lard_, post-HFD_lard_). TSE metabolic phenotyping cages were used during the change to HFD at 16 weeks old (pre- and post-HFD), an ITT post-HFD was performed at 20 weeks old, and resting blood glucose was taken at 24 weeks old.

### Metabolic phenotyping: procedures

#### Insulin tolerance test

Each mouse underwent 1 week of habituation to daily handling and mock i.p. administration to minimize the stress induced by handling. Cages were changed the evening before the ITT to provide consistently clean cages and minimize leftover food spilling into the bedding. ITTs were performed in ad libitum-fed mice 2 h into the light cycle and food was withdrawn from the cage at the beginning of each test. Following determination of body weights and basal blood glucose concentrations, mice received an i.p. injection of 0.75 U kg^−1^ body weight of human insulin (Insuman Rapid, Sanofi Aventis) dissolved in room temperature 0.9% saline (Berlin Chemie). Blood glucose concentrations were measured from venous tail blood at baseline, 15, 30 and 60 min after injection using an automatic glucose monitor (Contour Ascensia, Bayer HealthCare).

#### Body composition analysis

Body composition analysis was performed using micro-computed tomography (micro-CT)-based imaging of mice under isoflurane anaesthesia. Data acquisition was performed in an IVIS Spectrum CT-scanner (Caliper LifeScience) using the IVIS LivingImage Software v.4.3.1. Quantification of fat and lean mass was performed with a modification of the Vinci software package v.4.61.038. Lean and fat mass were normalized to 100% before statistical analysis.

#### ELISA

Circulating leptin, CCK and PYY levels were measured in ad libitum-fed dams 5 h into the light phase. Circulating insulin levels were measured in overnight-fasted mice. For refeeding experiments, circulating leptin, CCK and PYY levels were measured in 12 weeks old overnight-fasted female mice. To perform experiments in HFD_lard_ non-naive mice, mice have received small pieces of HFD_lard_ pellets at least three times before experiments. Blood was collected by decapitation 10 min after HFD feeding. The blood was then centrifuged for 10 min at 10,000*g*, and the serum fraction was collected and snap frozen in liquid nitrogen. Insulin ELISA (Crystal Chem, 90080), leptin ELISA (Crystal Chem, 90030), PYY ELISA (Crystal Chem, 81501) and CCK ELISA (RayBiotech, EIA-CCK) were performed according to the manufacturer’s protocol.

#### HOMA-IR calculation

HOMA-IR was calculated as insulin (µIU ml^−1^) multiplied per glucose (mmol l^−1^) divided by 22.5.

#### Metabolic phenotyping cages

Measure of indirect calorimetry (EE and respiratory exchange ratio), locomotion and food intake were performed using the PhenoMaster System (TSE Systems). Mice were moved into training cages 3 days before data acquisition for acclimatation. Mice were maintained at 22 °C in sealed cages with ad libitum access to food and water. After 1 day in the PhenoMaster System to further acclimate, data acquisition was performed for 2 consecutive days starting 2 h into the light cycle on mice fed their respective diet (pre-HFD_lard_). Following the 2 days of pre-HFD_lard_ at 3 h into light cycle, mice were switched onto HFD_lard_ and recordings continued uninterrupted (post-HFD_lard_). All parameters were measured continuously and simultaneously. Data regarding EE were corrected for lean mass.

### Milk collection

At 12 days after giving birth, lactating mothers were injected with 2IU oxytocin (Merck, O4375), anaesthetized under isoflurane (2–3%), and maintained at 37.0 ± 0.5 °C by a thermostatically controlled water heating system. Then, 5 min later the nipples were manually massaged to promote milk ejection, which was collected manually using a pipette. The milk was immediately snap frozen in liquid nitrogen for storage at –80 °C until analysis. Dams were killed after milk collection and tissue collected from offspring.

### Amniotic fluid collection

Timed-pregnant dams carrying E15.5 fetuses fed NCD or BFD as described above were anaesthetized using 2% isoflurane and maintained on 2% isoflurane during the procedure. The abdominal skin was sterilized with Octenisept (Schülke) and an abdominal incision was made. The uterine horns were gently placed outside the abdominal cavity, sectioned and placed in phosphate-buffered saline (PBS) solution. Individual amniotic sacs were exposed and placed in a dry, sterile Petri dish. The amniotic membrane was carefully opened, allowing amniotic fluid to leak into the Petri dish, which was collected using a filtered micropipette and snap frozen in liquid nitrogen for storage at –80 °C until analysis. Following amniotic fluid collection, fetal and placental weight were recorded. Dams were killed after amniotic fluid collection and tissue collected from offspring.

### Maternal behaviour

Maternal behaviour was assessed at P5. Dams were transported in their home cage to the test room 1 h before the experiment for acclimatization and remained in their home cage throughout the behavioural test. Trials were conducted between 4–7 h into the light phase. A camera (C920s HD Pro Webcam camera, Logitech) was mounted to capture a top-down view and connected to a laptop for recording. Each dam completed six trials, alternating between male and female pups (three males and three females). For each trial, one pup along with a small amount of bedding was placed on a heating pad. Once the dam returned to the nest, the pup was placed at the diagonally furthest point from the nest. Each trial lasted a maximum of 2 min. Analysis was conducted by measuring the time (in sec) required to retrieve the pup to the nest.

### Gene expression analysis

#### Gene expression analysis

Samples were collected and snap frozen in liquid nitrogen. messenger RNA was extracted from the tissue using the mirVana miRNA Isolation kit (Ambion) and cDNA was produced by reverse transcription (High-capacity cDNA Reverse Transcription kit, Applied Biosciences). qPCR was performed using TaqMan Universal PCR Master Mix (Thermo Fisher) with Taqman probes (Thermo Fisher) listed below on a QuantStudio 7 Flex Real-Time PCR System (Applied Biosystems). *Gapdh* (Mm99999915_g1) was used as a housekeeping gene unless otherwise stated and data were analysed using the ΔCt method.

#### Dam hypothalamus

At weaning of the pups (P21) ad libitum-fed dams were killed by decapitation. Taqman probes used: *Agrp* (Mm00475829_g1), *Npy* (Mm00445771_m1), *Pomc* (Mm00435874_m1), *Cartpt* (Mm04210469_m1), *Sst* (Mm00436671_m1), *Hcrt* (Mm01964030_s1), *Crh* (Mm01293920_s1), *Trh* (Mm01182425_g1), *Prl* (Mm00599949_m1), *Oxt* (Mm01329577_g1) and *Gal* (Mm00439056_m1).

#### iBAT

Adult females’ offspring ad libitum-fed were killed by decapitation after 1 week of HFD_lard_ feeding. Taqman probes used were *Ucp1* (Mm01244861_m1), *Adrb3* (Mm00442669_m1), *Cidea* (Mm00432554_m1), *Pparg* (Mm01184322_m1) and *Ppargc1* (Mm01208835_m1).

#### Tongue

Adult offspring were killed by decapitation. Taqman probes used were *Cd36* (Mm00432403_m1), *Ffar4* (Mm00725193_m1) and *Plcb2* (Mm01338057_m1).

#### Gene expression analysis following neonatal optogenetic stimulation

Cre-negative and OMP-Cre-positive offspring heterozygous for ReaChR were light-stimulated or mock-stimulated once at P3 and returned to their home cage with their mother for 1 h until killing by decapitation. Taqman probes used were *Egr1* (Mm00656724_m1), *Sema7a* (Mm00441361_m1), *Pcdh10* (Mm00477987_s1), *Cnga4* (Mm01278645_m1), *Neurog1* (Mm00440466_s1), *Adcy3* (Mm00460371_m1) and *Omp* (Mm00448081_s1). *Omp* was used as a housekeeping gene.

### HFD odour-induced hepatic p-mTOR

#### Olfactory exposure in freely behaving mice

Mice were split into cages containing two mice each 1 week before the experiment. A closed perforated metal tea ball was placed into their cages 3 days before to reduce the impact of a novel object during the experiment. Mice were acclimated to the experimental rooms and daily handling to minimize the effects of the odours related to the experimental room and the experimenter. After the acclimatization period, mice were fasted overnight and 2 h into the light cycle exposed to an empty tea ball (control, no odour) or to three pellets of HFD_lard_ in a tea ball that allowed mice to smell HFD_lard_ odours without physical access to the diet. Then, 30 min later, the mice were decapitated and the liver was quickly dissected and snap frozen in liquid nitrogen.

#### Protein isolation and western blotting

Approximately 50 mg liver tissue was transferred to a 2-ml tube containing 1 ml lysis buffer (containing Tris-HCl 25 mM, NaCl 25 mM, NP-40 1%, EDTA 1 mM, pH 7.4) with freshly added Halt Protease and Phosphatase Inhibitor Single-Use Cocktail (Thermo Fisher Scientific) and about 20 ZiO_2_ beads (VWR, CAS 432-0356). Samples were shaken for 2 min or until the tissue was fully digested. Samples were then centrifuged for 20 min at 15,000*g* and supernatant taken for protein quantification using Pierce BCA assay (Thermo Fisher Scientific). The protein samples were diluted to 2 mg ml^−1^ and mixed 1:4 with Laemmli sample buffer containing 10% β-mercaptoethanol.

Protein samples were run on a precast gel from Bio-Rad (4–15%) with 26 wells. Gels were run with a constant current of 130 V for 80 min. Transfer of gels to PVDF membranes was performed using Trans-Blot Turbo System (Bio-Rad) at 2.5 A and 25 V for 13 min. Gels were run in running buffer containing 25 mM Tris-HCl, 190 mM glycine and 0.1% SDS. Blots were blocked in 5% Western Blocking Solution (Roche, 11829200) in TBS-T (20 mM Tris, 150 mM NaCl, 0.1% Tween-20, pH 7.4) for 1 h at room temperature. Blots were incubated in the same solution containing anti-phospho mTOR (S2448) (Cell Signalling, 2971S) overnight at 4 °C. The next morning, the membrane was washed 3 × 5 min in TBS-T and the secondary HRP-conjugated anti-rabbit IgG (Invitrogen, 31466) was incubated at 1:1,000 for 60 min. Proteins were imaged using the Fusion Solo Vilber Lourmat system. Afterwards, blots were stripped in stripping buffer (10% SDS, 0.5 M Tris, 3.5% β-mercaptoethanol, pH 6.8) for 30 min at 56 °C and membranes were washed with TBS-T for 3 × 10 min and re-blocked. Incubation with anti-mTOR (Cell Signalling, 2972S) and HRP-conjugated anti-rabbit IgG (Invitrogen, 31466) was repeated as before. Band density was measured with ImageJ (National Institutes of Health). Data are presented as p-mTOR/mTOR and normalized to the average ratio of the non-food odour-exposed animals.

### iBAT temperature

#### iBAT temperature upon food odour exposure

At 8 weeks old, an implantable electronic transponder with thermal couple (IPTT-300, Bio Medic Data Systems) was placed between the scapulae through a small incision under anaesthesia (2% isoflurane). The incision was allowed to heal for 1 week during which the animals were acclimated to handling and having temperature measurements taken with a wireless reader (DAS-8007C, Bio Medic Data Systems). The animals remained group housed. An empty tea ball was placed in the cages 3 days before the experimental day to eliminate effects of a novel object in the cage. On the day of experimentation, food was removed 2 h before the dark cycle. Baseline temperature measurements were taken every 20 min starting at the beginning of the dark cycle. Three readings for each mouse were averaged for the baseline value. A tea ball containing three pellets of HFD_lard_ was then placed into the cage 1 h into dark cycle and temperature readings were taken 20 min later. All readings were recorded using DASHost 8000 software (Bio Medic Data Systems).

#### Longitudinal measurements of iBAT temperature

The 6–9-week-old mice were acclimatized to handling and thermal imaging procedure for approximately 2–3 weeks before experiments. Mice were carefully imaged using a thermal camera (FLIR E6, Teledyne FLIR LLC) by placing the camera at approximately 10 cm from the interscapular region. Temperature readings were taken weekly with an initial baseline measurement (pre-HFD; 9–12 weeks old), followed 1 week later by a diet change to HFD_lard_ (1 h measurement) and 4-week follow-up measurements. Thermal images were analysed manually using thermal imaging software (FLIR Thermal Studio v.1.9.23.0).

### Neonatal non-invasive optogenetic stimulation

OMP-Cre or M71-IRES-Cre were crossed to R26-LSL-ReaChR-mCitrine to allow for the expression of the optogenetic channelrhodopsin in all OSNs or only in OSNs expressing M71, respectively. The breeding strategy was similar to the one described above except that all dams were fed NCD. To optogenetically stimulate developing OSNs, a fibre optic cable (200 µm diameter, 0.48 NA, Doric Lenses) was held directly above but not in contact with the snout of the pup to deliver light at 625 nm (LEDFLS_625_625LED Fibre Light source, Doric Lenses). The stimulation paradigm was rectangular pulses of 100 ms ON/100 ms OFF for 3 s, 12 s OFF, repeated 12 times at 250 mA. This protocol has been previously demonstrated to replicate activity-dependent wiring of olfactory circuits and the glomerular map^52^. OMP^ReaChR^ mice and their Cre-negative control littermates were stimulated once at P3 for gene expression analysis. M71^ReaChR^ and their Cre-negative control littermates were stimulated daily from P1 to P7. To minimize the stress induced by the manipulation, the experimenter’s gloves were rubbed with the bedding of the home cage to carry home cage and maternal odorants, and neonatal optogenetic stimulation was performed on a warm heating pad.

### Neonatal non-invasive optogenetic stimulation coupled with calorie ingestion

The same paradigm as described above for neonatal non-invasive optogenetic stimulation was followed, with the modification that the pups were fed an Ensure liquid diet (Abbott, 640587) during stimulation. The Ensure diet was freshly prepared each day and warmed to 37 °C before being offered to the pups. 100 μl of Ensure was placed on a heating pad and pups were gently placed and kept close to it during light stimulation. All pups consumed Ensure during the experiments.

### Neonatal optogenetic activation induced pS6 activation

Mice were optogenetically stimulated following the same paradigm described above. Mice were stimulated at P5 and then returned to the cage with the dam. At 1 h later, P5 pups were killed by decapitation. Heads, were quickly rinsed with 0.1 M PBS, followed by fixation in 4% PFA for 24 h. Then, they were transferred into 20% sucrose in PBS for 48 h, followed by being embedded in tissue freezing medium (14020108926, Leica Biosystems). After being frozen they were cut at 30 μm on a cryostat (Leica). For the immunostaining, slides were blocked with 5% donkey serum (diluted in 0.3% Triton and 1× PBS) for 1 h at room temperature, and then incubated with the primary antibody anti-GFP (1:500 dilution, ab13970, Abcam) and anti-pS6 (Ser244, Ser247) (1:1,000 dilution, 44-923G, Thermo Fisher Scientific), followed by incubation in the secondary antibody anti-rabbit IgG (1:500 dilution, A11012, Thermo Fisher Scientific). Tissue was coverslipped with Vectashield mounting solution with DAPI (Biozol VEC-H-1200, Vector Laboratories). Images were acquired using a confocal microscope Leica STELLARIS (Leica Microsystems) with a ×40 objective.

### Behavioural tests

#### Diet preference tests

##### Adult C57BL/6NCrl cohorts

The 7-week-old C57BL/6NCrl mice were single housed for 1 week and acclimated to two food hoppers (TSE Systems) for 4 days. Following the acclimation period, baseline food intake was measured for 3 days. The baseline food intake depicted in the figure represents the average daily food intake during these 3 days. The following day, mice were switched on HFD_lard_ versus BFD or NCD versus BFD shortly before the onset of the dark cycle. Food intake was monitored for 5 days. For representation, baseline NCD intake is plotted as the average of the 3 days of NCD measurements.

##### NCD_dev_ and BFD_dev_ cohorts

At 8 weeks old, mice were single housed for 1 week and acclimated to two food hoppers allowing manual weighing of food intake. Following the acclimation period, on day 1, the diets in the food hoppers were changed to one food hopper containing HFD_lard_ and the other containing CD, novel for both groups. For three consecutive days, food intake was recorded once per day after the onset of the light cycle and converted to calories of diet consumed. Data were calculated as percentage of calories consumed from HFD_lard_ over the control diet.

##### Longitudinal food intake measurements

At 7 weeks old, mice were single housed for 1 week to acclimate to the food hopper and water bottle of the PhenoMaster System (TSE Systems). Mice had ad libitum access to food and water at all times. At 8 weeks old, mice were switched to HFD_lard_ and food intake was measured until 20 weeks of age. Food intake was measured daily for the first 2 weeks on HFD and biweekly from week 2–10 on HFD.

##### Latency to eat

At 6 weeks old, the latency to eat was measured in overnight-fasted mice to determine relative attraction or aversion to AFD. Approximately 3 h into the light cycle, mice were placed individually into a clean cage and allowed to acclimate for 2 min. A single pellet of either NCD or AFD was then placed into the cage and the time it took for the mouse to approach and take the first bite of the pellet was measured. Mice were exposed to NCD or AFD in two consecutive weeks. The latency to eat AFD was normalized to the latency to eat NCD.

##### Odour investigation test

Approximately 3 h into the light cycle, ad libitum-fed mice were placed into a clean cage with a closed lid and allowed to acclimate for 2 min. Cotton swabs were dipped in solutions containing either vehicle (10% dimethylsulfoxide (DMSO) in water) or progressively increasing concentrations of acetophenone. The cotton swab was inserted through the hole for the water bottle spout to not open the cage between trials. The time spent sniffing each concentration, defined as head tilted up towards the cotton swab performing active sniffing motions, was measured over 2 min. The relative ratio of time spent sniffing each concentration of acetophenone normalized to time spent sniffing the vehicle was quantified for each group.

##### Habituation-dishabituation test

Approximately 3 h into the light cycle, ad libitum-fed adult female mice were moved into the experimental room and individually placed into a clean cage with a closed lid and allowed to acclimate for approximately 15 min. Mice were exposed to a sequential presentation of different odours presented in a perforated metal tea ball: empty tea ball (control), NCD, 1% AFD, 0.25% AFD and HFD_lard_. Each odour is presented in three consecutive trials for a period of 2 min, with a 15–20-s intertrial time. The time spent sniffing each odour was defined as the head pointed up and active nose/whisker movement. Quantification was based on video analysis taken from the side view.

### Stereotaxic surgery

#### Dopamine and calcium fibre photometry

All surgeries were performed in 8 weeks old mice. Mice were anaesthetized using 2% isoflurane and placed in a stereotaxic apparatus (Kopf Instruments). Before exposing the skull, the skin was sterilized with Octenisept (Schülke) and Anesderm (Pierre Fabre) was applied to the incision site. A small hole was drilled above target sites for viral delivery and fibre implantation. A 0.5-µl Neuros Hamilton Syringe (Hamilton 65450-03) was used to inject the virus at a rate of 100 nl min^−1^ and left in place for 5 min before being slowly withdrawn.

For photometry recordings, AAV9.Syn.Flex.GCaMP6s.WPRE.SV40 (400 nl, a gift from D. Kim & GENIE Project, Addgene viral prep #100845-AAV9) or AAV5-CAG-dLight1.1 (400 nl, a gift from L. Tian, Addgene viral prep #111067-AAV5) viruses were injected in the ARH and LAcbSh, respectively, using the following coordinates relative to bregma (in mm): ARH: −1.45 AP, 0.25 ML, −5.75 and −5.85 DV; LAcbSh: 1.0 AP, 1.75 ML, −4.6 and −4.7 DV. After virus delivery, an optical fibre (400 µm diameter, 0.48 NA, Doric Lenses) was implanted directly above the site of injection and secured using dental acrylic (Super-Bond C&B, Sun Medical). For postoperative care, mice were administered buprenorphine (0.1 mg kg^−1^, i.p.) and meloxicam (5 mg kg^−1^, subcutaneously) and received tramadol in the drinking water (1 mg ml^−1^) for 2 days before and 3 days after surgery.

#### M71^ReaChR^_adult_ optogenetic stimulation

The 12-week-old M71-IRES-Cre:R26-LSL-ReaChR-mCitrine mice were anaesthetized, placed in the stereotaxic apparatus and subsequently implanted with an optical fibre (200 µm diameter, 0.48 NA, Doric Lenses) targeting the right M71 glomeruli in the olfactory bulb, using the following coordinates relative to bregma (in mm): 4.1 AP, 0.25 ML and 1.0 DV.

### Dual-colour fibre photometry

#### Data collection

Data were acquired using a RZ5P lock-digital processor controlled by Synapse software (Tucker-Davis Technologies) as described previously^84^.

#### Recording: dopamine sensor

Fibre photometry recording started 5 weeks after surgery to allow for mice recovery and optimal viral expression. Here, 2 weeks after surgery, mice were acclimated to the patch cord in the relevant behavioural setup for about 15 min each day. Mice were fasted overnight once during the fourth week post-surgery to reduce the future effects of first-time fasting. During the fifth week post-surgery, mice were fasted overnight and placed into a small experimental box 2–4 h into the light cycle. The experimental box (interior dimensions, 11.5 × 14 ×12 cm) is made in transparent plexiglass (wall thickness: 0.9 cm) with a circular cutout in the top corner (2 cm diameter) and a sliding lid containing a small hole to allow for the fibre optic cable to pass through. The experimental box was placed in a much larger arena with opaque black walls to block any view of the experimenter outside. A tube ran from outside the larger arena and through the circular cutout of the smaller box to allow for pellets to be delivered without interference from the experimenter. Food pellets were approximately ~20 mg each. After 10 min of acclimation, small pellets of BFD were provided to the mice at random intervals between 1 and 3 min to prevent anticipatory responses. An additional pellet was not given if the mouse had not eaten an already present pellet or if the mouse sat just below the delivery tube. In total, five pellets were delivered, as further pellets showed diminished responses during testing. Mice with no dopaminergic responses to refeeding on BFD were excluded from future experiments and analysis. This paradigm was repeated with 1 week in between for NCD and HFD_lard_. Mice were provided with a small pellet of HFD_lard_ a few days before the trial to eliminate artifacts related to the novelty-induced neophobia.

#### Recording: calcium recording of AgRP neurons

Fibre photometry recording started 4 weeks after surgery to allow for mouse recovery and optimal viral expression. Mice were kept on their respective diet (pre-HFD_lard_) until 15 weeks of age and then both groups were switched to HFD_lard_ (post-HFD_lard_). Starting 2 weeks after surgery, mice were acclimated to the patch cord in the relevant behavioural setup for about 20 min each day. At 3–4 weeks post-surgery, mice were placed into a clean cage without bedding and allowed to acclimate for 10 min. Mice were then injected i.p. with ghrelin (50 μg; Tocris 1465) diluted in 0.9% saline and the signal was recorded for 5 min. Mice with no changes in 465 nm fluorescence signal in response to ghrelin were excluded from future studies.

#### AgRP neuronal response to diets

At 5 weeks post-surgery, mice were then fasted overnight and placed in the photometry recording setup at 2–4 h into the light cycle. Following a 10 min baseline recording, a pellet of either NCD or HFD_lard_ was placed into the cage and signal was recorded for 5 min. At 7 weeks post-surgery (at 15 weeks old), mice were switched to HFD_lard_ in their home cage (post-HFD_lard_). At 3 weeks post-HFD_lard_, mice were fasted and recordings were repeated with NCD exposure. 1 week later, overnight fasting was repeated and recording was performed during HFD_lard_ exposure.

#### AgRP neuronal response to hormones

AgRP neuronal fibre photometry recordings started 4 weeks post-surgery (after 1 week of acclimatation to the cable and behavioural setup). For all recording of AgRP activity dynamics in response to hormones mice were tested every week following the same experimental design: mice were placed in the behavioural setup and allowed to acclimate for 10 min before i.p. injection of a hormone and the signal was recorded for 20 min. At 12 weeks (4 weeks post-surgery), mice were injected i.p. with ghrelin (2 mg kg^−1^ BW; Tocris 1465). At 13 weeks, mice were fasted overnight, placed in the recording setup and injected i.p. with PYY (0.1 mg kg^−1^ BW; R&D Systems). After the recording session, animals were returned to their home cages and given ad libitum access to food. At 14 and 15 weeks, mice were fasted overnight and tested as described above for serotonin hydrochloride (2 mg kg^−1^ BW; Sigma-Aldrich) and glucagon (2 mg kg^−1^ BW; Bachem) respectively. All hormones were diluted in 0.9% saline and injected with a volume of 10 µl g^−1^ BW.

#### Viral expression and fibre placements

Virus expression and fibre placement were verified as described previously^85^.

### Fibre photometry data analysis

All fibre photometry data analyses were performed using a custom MATLAB script. In the event of technical issues encountered on a recording day, such as malfunctions with the photometry rig during the recording session, the animal was excluded from this experimental time point.

#### dLight analysis

The initial 10 s of recording (laser power-up) was discarded and the data were downsampled at 50 Hz. To correct for artifactual signal fluctuations, the 405 nm Ca^2+^-independent isosbestic reference was aligned to the 465 nm signal using least-squares linear fit and then used as a baseline to compute the ΔF/F = (465 nm – scaled 405 nm)/(scaled 405 nm). The peri-event trace was extracted using a window spanning −30 s to +60 s around the moment of approach to the pellet manually identified from video recordings. Each trial was then *z*-scored using the −30 s to −20 s window as baseline to calculate the mean and s.d. All trials of a single animal and single diet were averaged, and the mean (*z*-scored) trace between 0 s to +5 s and +5 s to +10 s were taken to compare groups using unpaired *t*-test.

### AgRP photometry analysis

The initial 10 s of recording was discarded and the data were smoothed using a 1-s moving-average window before being downsampled at 50 Hz. Peri-event traces were extracted from a window spanning −60 s to +180 s around the food pellet introduction or −120 s to +180 s around ghrelin injection. To avoid spurious influences of the stimulus administration, the baseline was defined as the window from −60 s to −20 s for food pellets and from −120 s to −60 s for CCK injection. For analysis of AgRP neuronal responses to ghrelin, PYY, 5-HT and glucagon, a similar approach was used with a smoothed using a 3-s moving-average window and downsampled at 50 Hz. Data were extracted from a window of −300 s to +1,200 s around the moment of injection. Baseline was defined from the window of −300 s to −60 s. The 465 nm signal was then *z*-scored, using this baseline to calculate the mean and s.d. Each trial was then *z*-scored using this baseline to calculate the mean and s.d. The mean (*z*-scored) trace between +15 s and +180 s was computed for each animal and then used to compare groups via unpaired *t*-tests.

### M71 photostimulation-generated odour preference

The experiment followed the protocol for M72 photostimulation-generated odour preference described by Vetere et al.^86^, with minor modifications.

#### Odorant preparation

An M71-activating odorant (acetophenone; A10701, Sigma-Aldrich) or a non-M71-activating odorant (isoamyl acetate; 101231, Merck) were diluted in mineral oil (330779, Sigma-Aldrich) achieving a 40% concentration. The mixtures were stored in light-protected vials. To use the odorants, 50 μl of an odorant was pipetted onto filter paper on an inverted Petri dish (60 × 15 mm) and covered with cage bedding.

#### M71-glomerulus photostimulation protocol

The delivery of light pulses (625 nm, 85 mA, 4 Hz, 100 ms ON, 150 ms OFF) was designed to approximately resemble mice’s average natural duration of a sniff (100 ms) and breathing frequency (3–5 Hz)^87–92^.

#### Behavioural paradigm

All mice were naive to HFD_lard_, sucrose, acetophenone and isoamyl acetate before the experiment. The behavioural paradigm consisted of three phases: pre-exposure (day 1), optogenetic conditioning (day 2) and odour preference test (day 3). The experiment took place in a custom-designed preference test chamber made of white Plexiglas (15 × 40 × 25 cm) divided into two identical compartments by removable walls. On the first day, mice were plugged to the patch cord and underwent a 25-min pre-exposure to the test chamber, with each chamber compartment containing a non-scented Petri dish filled with bedding material. Mice did not receive any photostimulation during this first day. Then, mice were fasted overnight (16 h). On the second day, mice were exposed to a 25-min conditioning period. After the removable walls of the Plexiglas chamber were taken out, fasted mice were plugged to the patch cord and exposed to a non-scented Petri dish located in the centre of the chamber. The bedding in this Petri dish contained small pieces of HFD_lard_ coated with 40% sucrose, which had been prepared the previous day. All mice engaged with the food pellets and ingested them. Simultaneously, mice received M71 photostimulation constantly during the 25 min of the conditioning, as described above. On the third day, the removable walls were re-inserted, and a Petri dish scented with either acetophenone or isoamyl acetate was placed into one of the chamber compartments, the location of which was randomly assigned. Mice were allowed to freely explore both compartments for 10 min without receiving any photostimulation. Mouse behaviour was continuously monitored with an overhead camera. These results reflect the whole 10-min observation period. Odour preference during the test day was calculated using the following formula: percentage of time spent in the acetophenone compartment – percentage of time spent in the isoamyl acetate compartment. After the experiments, mice were deeply anaesthetized with ketamine/xylazine and perfused transcardially with 0.1 M PBS followed by 4% PFA (pH 7.4) for future processing to verify fibre placements.

### Histology

#### Tissue processing

For fibre photometry experiments, mice were deeply anaesthetized with ketamine/xylazine and transcardially perfused with 4% PFA in 0.1 M PBS, pH 7.4. Tissues were post-fixed overnight in the same PFA solution. Brain tissues from photometry experiments were cryoprotected in 20% sucrose in PBS and cut at 30 μm on a cryostat (Leica). Tissue was coverslipped with Vectashield mounting solution with DAPI (Biozol VEC-H-1200). Sections were imaged using only the endogenous fluorescence of GCaMP6s or dLight1.1. White adipose and liver tissues were post-fixed for 24 h, dehydrated in 30% sucrose and embedded in paraffin before cutting at 5 μm on a vibratome and directly mounted. Tissue was stained with hematoxylin (GHS132, Sigma-Aldrich) and eosin (HT110232, Sigma-Aldrich) after deparaffinization.

#### Microscopy

All tissue was imaged on a Zeiss Imager M2 microscope and AxioVision v.4.2 software (Carl Zeiss) at ×20 magnification.

#### Quantification

Adipocyte size was quantified using ImageJ software for a minimum of 500 adipocytes per mouse from five randomly selected areas.

### Ribosome immunoprecipitations (ps6-ribotrap)

#### Tissue preparation and ribosome immunoprecipitation

Phosphorylated ribosome pulldown for the identification of olfactory receptor activation was adapted from previous protocols^31^. Experiments were performed on males C57Bl6/NCrl from 8–12 weeks old of age. Following 1 h of odour exposure, the olfactory epithelium was dissected on ice in buffer containing 1×HBSS (Gibco, with Ca^2+^ and Mg^2+^), 2.5 mM HEPES (pH 7.4), 35 mM glucose, 100 μg ml^−1^ cycloheximide, 5 mM sodium fluoride, 1 mM sodium orthovanadate, 1 mM sodium pyrophosphate and 1 mM β-glycerophosphate. Four epithelia were pooled and processed as described previously^85^.

#### RNA sequencing

Due to the low amount of input material, RNA-seq libraries were prepared using the Ovation RNASeq System V2 and Illumina Nextera XT protocol, as previously described^93^, and sequenced on an Illumina HiSeq 4000 (2 × 75 bp). We applied the community-curated nfcore rnaseq analysis pipeline v.1.446. The gene-level quantification was carried out using Salmon v.0.14.147 using the reference genome GRCm38. To normalize the immuno-pulldown for each sample to its background, we divided the Salmon gene count per sample using the formula countpulldowncountinput. The differential gene expression analysis based on the normalized counts was carried out using the DESeq2 v.1.26. R package^94^. Gene Ontology term analysis was carried out using the clusterProfiler v.3.14.3 R package^95^. Olfactory receptors with a *P*_adj_ < 0.001 compared with no odour samples were defined as significantly activated, as defined by Jiang et al.^31^.

### Two-phase metabolite extraction of polar and lipophilic metabolites (dams milk, blood and food pellets)

For the extraction of total lipids, 50 µl milk/plasma were collected in 2-ml round bottom Eppendorf tubes. For the extraction of the snap frozen material, the milk/plasma samples were allowed to thaw on ice. Lipids were then extracted by adding 1 ml pre-cooled (−20 °C) extraction buffer containing internal standard as described previously^96^.

#### LC–HRMS-based analysis of dams milk and blood lipids

The dried lipid extracts were resuspended in 200 µl UPLC-grade acetonitrile:isopropanol (70:30 (v:v)) before analysing them on a UPLC connected to a Tribrid Orbitrap HRMS as described previously^96^.

The obtained Thermo .raw files were converted to .mzXML files using MSConvert from ProteoWizard software package (http://proteowizard.sourceforge.net)^97^.

#### Data pre-processing for untargeted lipidomics analysis

Data pre-processing was performed with MZmine v.2.340 (http://mzmine.github.io/)^98^. Converted mzXML files were imported and extracted ione chromatograms were generated using the ADAP algorithm and peak picking was performed using the local minimum search algorithm. Isotopes were grouped and only peaks with at least one isotope were kept for further processing. Detailed parameters can be found in Supplementary Table 5. Finally, aligned peak lists were exported to CSV files for statistical analysis. Tables were corrected for solvent peaks by a blank subtraction. Features were removed when average peak area of samples was less than 2× average peak area of blanks.

#### Data evaluation

For data evaluation, CSV files were imported in Perseus (http://www.biochem.mpg.de/5111810/perseus)^99^. Data were further filtered for features that had a coverage of at least 70% valid data in at least one group. Filtered data were log_2_ transformed and missing values were imputed from normal distribution using the default settings.

### LC–MS analysis of lipid extracts from food pellets

Lipidomics of food pellets was performed using a Thermo Vanquish Flex with a quaternary pump (Thermo Fisher Scientific) coupled to a TimTOF Pro2 mass spectrometer equipped with a heated ESI source (VIP-HESI) (Bruker Daltonics). Dried lipid samples were resuspended in 500 μl of acetronitril:isopropanol (70:30 (v:v)). The resuspended samples were cleared by a 5 min centrifugation at 16,000*g* and the 200 µl of the supernatants were transferred to 2-ml glass vials with 300 µl glass inserts (Chromatography Zubehör Trott). Then, 1 µl was injected in the UHPLC system and separated as described above. Compounds were detected in the mass spectrometer using data-dependent parallel accumulation serial fragmentation (DDA-PASEF) acquisition mode. All analysis were run in positive ionization mode using SA capillary voltage set to 4,500 V with an end plate off set of 500 V. Nebulizer pressure was set to 2 bar, dry gas was running with 8 l min^−1^ and a temperature of 230 °C. Sheet gas was running with 4 l min^−1^ and a temperature of 400 °C. For MS2 experiments masses were isolated with a width of 2 mD and fragmentation was induced with a collision energy of 30 eV.

### LC–HRMS-based analysis of polar and semi-polar compounds

The stored (−80 °C) polar and semi-polar extracts were resuspended in 100 µl of a UPLC-grade water: methanol (50:50 (v:v)). From each analytical sample a volume of 20 µl was taken and pooled together. These pools were used as instrumental and sample stability quality controls (QCs), which are run after every tenth analytical sample in the sample sequence or after each replicate group. All samples and pools were placed in an Vanquish flex UHPLC (Thermo Fisher Scientific), which was connected to a TimsTOF Pro 2 HRMS, (Bruker Daltonics). Of each sample 1 µl was injected onto a 100 × 2.1 mm HSS T3 UPLC column, packed with 1.7-µm particles (Waters). The flow rate of the UHPLC was set to 500 μl min^−1^ and the buffer system consisted of buffer A (0.1% formic acid in UPLC-grade water) and buffer B (0.1% formic acid in UPLC-grade acetonitrile). The UHPLC gradient was as follows: 0–1 min 100% A (curve = 6), 1–11.5 min 100–0% A (curve = 7), 11.5–12 min 0% A, 12.0–12.1 min 0–100% A, 12.1–15 min 100% A. This leads to a total runtime of 15 min per sample. For better annotation some quality pool sample injections were run at the end of the sequence with a data-dependent parallel accumulation serial fragmentation (DDA-PASEF) acquisition mode with the same stepped mobility ramp to generate a MS2 spectra collection. Source parameters were as follows. Capillary voltage was set to 4,500 V with an end plate off set of 500 V. Nebulizer pressure was set to 2 bar, dry gas was running with 8 l min^−1^ and a temperature of 230 °C. Sheet gas was running with 4 l min^−1^ and a temperature of 400 °C. For MS2 experiments masses were isolated with a width of 2 mD and fragmentation was induced with a collision energy of 20 and 50 eV.

#### Feature extraction and data analysis

All samples were analysed in a randomized run-order after the column was conditioned with several blank and some QC sample injections. Before and after the set of samples pooled QC sample were injected. Lipidomics analysis and untargeted reversed phase analysis of food pellets were both performed with the following criteria: Raw data was further processed with MetaboScape (v.2024b) to generate an untargeted feature table. Detailed processing parameters can be found in Supplementary Table 3. Filtered feature table were normalized to the TIC median and further submitted to MetaboAnalyst (v.6.0). Missing values were replaced by LoDs (one-fifth of the minimum positive value of each variable). Remaining features (lipidomics 4084; untargeted RP 4020) were log-transformed and pareto scaled before further analysis.

### Semi-targeted liquid chromatography-high-resolution mass spectrometry-based analysis of amine-containing metabolites

The LC–HRMS analysis of amine-containing compounds was performed using a QE-Plus high-resolution mass spectrometer coupled to a Vanquish UHPLC chromatography system (Thermo Fisher Scientific). Dried sample extracts were resuspended in 150 µl LC–MS-grade water for 10 min at 4 °C in a shaker at 1,500 rpm. After centrifugation, 50 µl of the extracts were mixed with 25 µl 100 mM sodium carbonate (Sigma), followed by the addition of 25 µl 2% (v/v) benzoylchloride (Sigma) in acetonitrile (UPC/MS-grade, Biosove), as reported previously. The derivatized samples were thoroughly mixed and kept at a temperature of 20 °C until LC–MS analysis^96^.

The LC–MS data analysis was performed using the open-source software MZmine 2 using MSConvert (2) (v.3.0.22060, Proteowizard). Compounds were annotated with an in-house library with an *m*/*z* tolerance of <5 ppm.

### VOC analysis

Analysis was performed in the laboratories of GC–ToFMS Sensenet (Odournet).

### Sample preparation

#### Diets

Approximately 4 g of diet were introduced into an individual microchamber and heated to 30 °C. A thermodesorption tube (Tenax/Carbocarph) was inserted in the microchamber to collect a total volume of 1,000 ml of headspace.

#### Milk and amniotic fluid

A total of 1 μl of milk or amniotic fluid was directly spiked in a desorption tube (Tenax). The milk and amniotic fluid samples used for the analyses consisted of a pool of 4–5 dams.

#### Analytical methods

Combined thermal-desorption gas chromatography and time-of-flight mass spectrometry TD-GC–ToFMS system was used to generate a full quantitative scan of VOCs from the previously collected samples. Analyses were performed using a thermal desorption unit (Unity, Markes International), a gas chromatographer (7890, Agilent), a time-of-flight mass spectrometer (BenchTOF-dx model, Almsco) and a mid-polar DB-624 column was used for chromatographic separation (60 m, 250 μm, 1.4 μm; Agilent). Desorption tubes were heated to 300 °C with a helium flow rate of 50 ml min l^−1^ for 10 min (first desorption stage). Desorbed analytes were directed to a hydrophobic general purpose cold trap (10 °C, thermoelectric cooling), filled with Tenax TA and graphitized carbon. After flash-heating of the cold trap to 320 °C during 5 min (second desorption stage), analytes were injected into the chromatographic column for further separation, which took about 53 min. Molecules reaching the ToFMS detector were fragmented by electron impact ionization at 70 eV at a mass range of 28–330 amu. Deuterated toluene-d8 (Sigma-Aldrich) was used as an external standard for quantification. This compound was injected (10 ng) into an independent thermodesorption tube and was analysed by following strictly the same methodology as with the samples. Given the sensitivity of the method, two unused thermodesorption tubes were analysed as blanks, to exclude any potential contamination arising from these materials during the analysis. The deconvolution process for the chemical identification of the VOCs that were present in each analysed sample was carried out with the software TargetView v.3 (ALMSCO International). This algorithm identified the compounds of the chromatogram automatically based on an updated version of the NIST20 library. Chemical identifications were confirmed with at least 80% certainty. Results of each diet/sample were normalized as per cent for representation.

### PET imaging

PET imaging was performed using an Inveon preclinical PET/CT system (Siemens) as previously described^100^.

#### Olfactory exposure during PET imaging

Olfactory exposure was performed as described^85^.

#### PET analysis

The kinetic model and statistical analysis was performed as previously described^85,100^.

### Statistical analysis

Statistical analysis was performed with GraphPad Prism 8 unless otherwise stated. Student’s *t*-test, Wilcoxon test, multiple *t*-test, one-way analysis of variance (ANOVA), two-way ANOVA and three-way ANOVA were used when applicable. Data not representing a Gaussian distribution were analysed by nonparametric tests. Statistical data are represented in the figure legends. In brief, datasets comparing experiments in the same animal subjected to two different treatments were analysed for statistical significance using paired two-tailed Student’s *t*-test. Datasets with only two independent groups were analysed for statistical significance using unpaired two-tailed Student’s *t*-test. Datasets with more than two groups were analysed using a one-way ANOVA followed by a multiple-comparisons test. Datasets influenced by two independent factors were analysed using a two-way repeated-measures ANOVA, followed by Sidak’s or Tukey’s multiple-comparisons test or a mixed-effects model followed by a Tukey’s multiple-comparisons test in cases where data were missing at certain time points. Datasets subjected to two independent factors were analysed using two-way repeated-measures ANOVA followed by a Sidak’s multiple-comparisons test in case of missing values at some time points. Three-way (type III) ANOVAs were performed in R v.4.3.0. For between-subject designs, we used linear models fitted with the stats package and analysed with the car package; for repeated measures, we implemented linear mixed-effect models using the lmerTest package and its built-in ANOVA function. No statistical methods were used to predetermine sample sizes but our sample sizes are similar to those reported in previous publications. Data distribution was assumed to be normal but this was not formally tested. Data collection and analysis were not performed blind to the conditions of the experiments. Alpha was defined as 0.05 and significance was depicted by *P < 0.05, **P < 0.01 and ***P < 0.001. Code used for the analyses is available upon request.

### Reporting summary

Further information on research design is available in the Nature Portfolio Reporting Summary linked to this article.

## Supplementary information

Reporting Summary

Supplementary Table 1 Raw data from dams’ blood analysis.

Supplementary Table 2 Raw data from milk metabolomics (untargeted analysis).

Supplementary Table 3 Raw data from food metabolomics (lipidomics analysis)

Supplementary Table 4 Raw data from food metabolomics (untargeted analysis)

Supplementary Table 5 Detailed parameters used for milk and blood lipid fraction analysis (related to Supplementary Fig. 3)

## Source data

Source Data Fig. 1 Statistical source data and unprocessed western blots.

Source Data Fig. 2 Statistical source data.

Source Data Fig. 3 Statistical source data.

Source Data Fig. 4 Statistical source data.

Source Data Fig. 5 Statistical source data.

Source Data Extended Data Fig. 1 Statistical source data.

Source Data Extended Data Fig. 2 Statistical source data.

Source Data Extended Data Fig. 3 Statistical source data.

Source Data Extended Data Fig. 4 Statistical source data.

Source Data Extended Data Fig. 5 Statistical source data.

Source Data Extended Data Fig. 6 Statistical source data.

Source Data Extended Data Fig. 7 Statistical source data.

Source Data Extended Data Fig. 8 Statistical source data.

Source Data Extended Data Fig. 9 Statistical source data.

Source Data Extended Data Fig. 10 Statistical source data.

## Data Availability

The RNA-seq data conducted in this study have been deposited in Gene Expression Omnibus under accession code: GSE308362. Source data are provided with this paper.
