# Supplementary material for: Fat sensory cues in early life program central response to food and obesity
Source: Nat Metab. 2025 Dec 1;7(12):2451–73. doi: 10.1038/s42255-025-01405-8 (PMC12727534; doi:10.1038/s42255-025-01405-8)
Supplement: Supplementary file 1 — Reporting Summary [file 42255_2025_1405_MOESM1_ESM.pdf]

nature portfolio

Corresponding author(s): Dr. Sophie Steculorum
Last updated by author(s): 30/04/2025

Reporting Summary

Nature Portfolio wishes to improve the reproducibility of the work that we publish. This form provides structure for consistency and transparency in reporting. For further information on Nature Portfolio policies, see our Editorial Policies and the Editorial Policy Checklist.

Statistics

For all statistical analyses, confirm that the following items are present in the figure legend, table legend, main text, or Methods section.

n/a	Confirmed	
☐	☒	The exact sample size (*n*) for each experimental group/condition, given as a discrete number and unit of measurement
☐	☒	A statement on whether measurements were taken from distinct samples or whether the same sample was measured repeatedly
☐	☒	The statistical test(s) used AND whether they are one- or two-sided<br>*Only common tests should be described solely by name; describe more complex techniques in the Methods section.*
☒	☐	A description of all covariates tested
☐	☒	A description of any assumptions or corrections, such as tests of normality and adjustment for multiple comparisons
☐	☒	A full description of the statistical parameters including central tendency (e.g. means) or other basic estimates (e.g. regression coefficient) AND variation (e.g. standard deviation) or associated estimates of uncertainty (e.g. confidence intervals)
☐	☒	For null hypothesis testing, the test statistic (e.g. *F*, *t*, *r*) with confidence intervals, effect sizes, degrees of freedom and *P* value noted<br>*Give P values as exact values whenever suitable.*
☒	☐	For Bayesian analysis, information on the choice of priors and Markov chain Monte Carlo settings
☒	☐	For hierarchical and complex designs, identification of the appropriate level for tests and full reporting of outcomes
☒	☐	Estimates of effect sizes (e.g. Cohen's *d*, Pearson's *r*), indicating how they were calculated

Our web collection on statistics for biologists contains articles on many of the points above.

Software and code

Policy information about availability of computer code

Data collection

- Indirect calorimetry and food intake data acquisition were carried out using TSE Phenomaster versions 6.2.5 and above.
- Body composition was measured using an IVIS Spectrum CT scanner (Caliper LifeScience, USA) using the IVIS LivingImage Software V4.3.1.
- Imaging of western blots was performed with FusionCapt Advance software (Vilber).
- Quantitative PCR was performed on a QuantStudio 7 Flex Real-Time PCR System using the QuantStudio Real-Time PCR Software v1.3 (Life Technologies).
- For ELISA, the optical density was determined using a FilterMax F5 Multi-Mode microplate reader and SoftMax Pro 6.3 software (Molecular Devices).
- For measurement of interscapular temperature, temperature measurements were taken using a wireless reader (Biomedic data systems DAS-8007C) or thermal camera (FLIR E6, Teledyne FLIR LLC)
- Liquid Chromatography-High Resolution Mass Spectrometry-based (LC-HRMS) analysis of lipids was performed using Thermo Fisher Q-Exactive GC Orbitrap GC-MS/MS with EI or CI source.
- PET imaging was performed using an Inveon preclinical PET/CT system (Siemens).
- Photometry recordings were performed using a RZ5P lock-digital processor controlled by Synapse software (Tucker-Davis Technologies, TDT, USA).
- Tissue was imaged on a Zeiss Imager M2 microscope and AxioVision 4.2 software (Carl Zeiss) or a Leica STELLARIS (Leica Microsytems)
- RNA-sequencing has been performed using Applied Biosystems 7900HT Sequence Detection and sequenced on an Illumina HiSeq 4000 sequencing instrument.
- Volatiles analyses were performed on a time-of-flight mass spectrometer (BenchTOF-dx model, Almsco,

Data analysis

Data analysis

- Data analysis was performed using Graphpad Prism (version 8.0; Graphpad Software Inc., La Jolla, CA, USA), unless otherwise stated below.
- All fiber photometry data analyses were performed using a custom Matlab script (version R2021a).
Three-way (type III) ANOVAs were performed in R 4.3.0. For between-subjects designs, we used linear models fitted with the stats package and analyzed with the car package; for repeated measures, we implemented linear mixed effect models using the lmerTest package and its built-in anova function.

- Analysis of PET Imaging was performed using the Vinci software.
- RNA-sequencing analysis was performed using Salmon 0.14.147 using the reference genome GRCm38. Analysis were performed using the DESeq2 1.26. R and the 3.14.3 R packages.
- Results of quantitative PCR experiments were calculated using Microsoft Excel.

- Densitometric analysis of protein bands was performed by FIJI (NIH, version 2.0.0-rc-41/1.50d).
- Quantification of fat mass (IVIS Spectrum CT scanner) was determined with a modification of the Vinci software package 4.61.038.
- The deconvolution process for the chemical identification of the VOCs that were present in each analyzed sample was carried out with the software TargetView v3 (ALMSCO International, Germany)
- Readings of interscapular temperature measurement were performed using DASHost 8000 software (Biomedic data systems).
- Analysis of LC-HRMS data was performed on Thermo RAW files were converted to mzXML files by MSConvert from Proteowizard 40. Further analysis were performed using MZmine 2.340 and Perseus.

For manuscripts utilizing custom algorithms or software that are central to the research but not yet described in published literature, software must be made available to editors and reviewers. We strongly encourage code deposition in a community repository (e.g. GitHub). See the Nature Portfolio guidelines for submitting code & software for further information.

Data

Policy information about availability of data

All manuscripts must include a data availability statement. This statement should provide the following information, where applicable:
- Accession codes, unique identifiers, or web links for publicly available datasets
- A description of any restrictions on data availability
- For clinical datasets or third party data, please ensure that the statement adheres to our policy

All raw data has been made available

Human research participants

Policy information about studies involving human research participants and Sex and Gender in Research.

Reporting on sex and gender

Use the terms sex (biological attribute) and gender (shaped by social and cultural circumstances) carefully in order to avoid confusing both terms. Indicate if findings apply to only one sex or gender; describe whether sex and gender were considered in study design whether sex and/or gender was determined based on self-reporting or assigned and methods used. Provide in the source data disaggregated sex and gender data where this information has been collected, and consent has been obtained for sharing of individual-level data; provide overall numbers in this Reporting Summary. Please state if this information has not been collected. Report sex- and gender-based analyses where performed, justify reasons for lack of sex- and gender-based analysis.

Population characteristics

Describe the covariate-relevant population characteristics of the human research participants (e.g. age, genotypic information, past and current diagnosis and treatment categories). If you filled out the behavioural & social sciences study design questions and have nothing to add here, write "See above."

Recruitment

Describe how participants were recruited. Outline any potential self-selection bias or other biases that may be present and how these are likely to impact results.

Ethics oversight

Identify the organization(s) that approved the study protocol.

Note that full information on the approval of the study protocol must also be provided in the manuscript.

Field-specific reporting

Please select the one below that is the best fit for your research. If you are not sure, read the appropriate sections before making your selection.

☒ Life sciences ☐ Behavioural & social sciences ☐ Ecological, evolutionary & environmental sciences

For a reference copy of the document with all sections, see nature.com/documents/nr-reporting-summary-flat.pdf

Life sciences study design

All studies must disclose on these points even when the disclosure is negative.

Sample size	No statistical methods were used to pre-determine sample sizes. Sample sizes in the study were chosen based on previous similar experiments performed in the laboratory or in the literature.
Data exclusions	For breeding paradigm, strict exclusion criteria were based on time to pregnancy and litter size (6-8 pups). For the photometry analyses, mice showing no viable fluorescent signals were excluded. All exclusion criterion were pre-established.
Replication	Every mouse represents a replicate (n) and the number of replicates is mentioned for each experiment. Experiments were performed in cohorts generated in different time periods.
Randomization	Mice have been assigned randomly to each group and experiment.
Blinding	Investigators were not blinded during group allocation or analysis due to the nature of the experiments and the presence of different diets in the cage.

Reporting for specific materials, systems and methods

We require information from authors about some types of materials, experimental systems and methods used in many studies. Here, indicate whether each material, system or method listed is relevant to your study. If you are not sure if a list item applies to your research, read the appropriate section before selecting a response.

Materials & experimental systems

n/a	Involved in the study	
☐	☒	Antibodies
☒	☐	Eukaryotic cell lines
☒	☐	Palaeontology and archaeology
☐	☒	Animals and other organisms
☒	☐	Clinical data
☒	☐	Dual use research of concern

Methods

n/a	Involved in the study	
☒	☐	ChIP-seq
☒	☐	Flow cytometry
☒	☐	MRI-based neuroimaging

Antibodies

Antibodies used	- anti-GFP (1:500, ab13970, abcam)<br>- anti-pS6 (Ser244, Ser247) (1:1000, # 44-923G, Thermo Fisher Scientific).<br>- anti-mTOR (1:1000, 2972S, Cell Signaling)<br>- HRP-conjugated anti-rabbit IgG (1:1000, 31466, Invitrogen)<br>- anti-phospho mTOR (S2448) (1:1000, 2971S, Cell Signaling)<br>- goat anti-rabbit IgG (1:500, A11012, Thermo Fisher)
Validation	anti-GFP (1:500, ab13970, abcam): (3612 publications) https://www.abcam.com/en-us/products/primary-antibodies/gfp-antibody-ab13970?srsltid=AfmBOorEamv23bU538PiV3vJ6gZAKgcLJE6xvTpRw5_pE71yIQvnffpu<br><br>anti-pS6 (Ser244, Ser247) (1:1000, # 44-923G, Thermo Fisher Scientific): (22 references) https://www.thermofisher.com/antibody/product/Phospho-S6-Ser244-Ser247-Antibody-Polyclonal/44-923G<br><br>anti-mTOR (Cell Signaling 2972S): (2033 citations) https://www.cellsignal.com/products/primary-antibodies/mtor-antibody/2972?srsltid=AfmBOopsHWtMgccOe55zJ00E9w5lPAWvEV1yC56o6aQApD6pv_5fCorP<br><br>HRP-conjugated anti-rabbit IgG (Invitrogen 31466): (171 references) https://www.thermofisher.com/antibody/product/Goat-anti-Rabbit-IgG-H-L-Secondary-Antibody-Polyclonal/31466<br><br>anti-phospho mTOR (S2448) (Cell Signaling 2971S): (2280 citations) https://www.cellsignal.com/products/primary-antibodies/phospho-mtor-ser2448-antibody/2971?srsltid=AfmBOopP0UQY3ncFBofR4RTfAmMY86s6eqafoqPuktHdHTziXkiYbV9P<br><br>secondary HRP-conjugated anti-rabbit IgG (Thermo Fisher A11012): (3598 reference) https://www.thermofisher.com/antibody/product/Goat-anti-Rabbit-IgG-H-L-Cross-Adsorbed-Secondary-Antibody-Polyclonal/A-11012

Animals and other research organisms

Policy information about studies involving animals; ARRIVE guidelines recommended for reporting animal research, and Sex and Gender in Research

Laboratory animals	C57BL/6N mice were purchased from Charles River (Germany). AgRP-IRES-Cre mice (JAX stock #012899), M71-IRES-Cre (JAX stock #006677), OMP-Cre (JAX stock #006668), and R26-LSL-ReaChR-mCitrine (JAX stock #026294) were originally obtained from The Jackson Laboratory. Cre lines were maintained heterozygous through breeding to wildtype C57BL/6N (Charles River, Germany), while R26-LSL-ReaChR-mCitrine mice were maintained as homozygous stock breeding at the Max Planck Institute for Metabolism Research, Cologne, Germany. All mice were housed in individually ventilated cages at 22-24°C at constant humidity (50-70%) with a 12/12-h light/dark cycle with ad libitum food and water unless otherwise stated for experimental conditions.
Wild animals	*Provide details on animals observed in or captured in the field; report species and age where possible. Describe how animals were caught and transported and what happened to captive animals after the study (if killed, explain why and describe method; if released, say where and when) OR state that the study did not involve wild animals.*
Reporting on sex	Metabolic phenotyping data in offspring exposed to BFD, PBFD and acetophenone during development have been generated in both genders. Hormonal release upon refeeding, iBAT thermogenesis and habituation-dishabituation tests have been performed in females only. Other experiments reported in the manuscript have been performed in male mice.
Field-collected samples	We did not use field-collected samples in this study.
Ethics oversight	Permission to maintain and breed mice was issued by the Department for Environment and Consumer Protection - Veterinary Section, Cologne, North Rhine-Westphalia, Germany.

Note that full information on the approval of the study protocol must also be provided in the manuscript.
